# Equine arteritis virus Nsp10 promotes MAVS proteasomal degradation via E3 ligases Smurf1/MARCH5

**DOI:** 10.1128/jvi.02061-25

**Published:** 2026-05-19

**Authors:** Bingqian Zhou, Kewei Chen, Haibing Liang, Ting Qi, Xing Guo, Yong-Jun Wen, Cheng Du, Xiaojun Wang

**Affiliations:** 1 State Key Laboratory of Animal Disease Control and Prevention, Harbin Veterinary Research Institute, Chinese Academy of Agricultural Sciences 111613 , Harbin, China; 2 Key Laboratory for Clinical Diagnosis and Treatment of Animal Diseases of Ministry of Agriculture, College of Veterinary Medicine, Inner Mongolia Agricultural University 117454 , Hohhot, China; 3 Institute of Western Agriculture, Chinese Academy of Agricultural Sciences 838119 , Changji, China; 4 China-Kazakhstan Joint Laboratory for Herbivorous Animal Disease Research, Heilongjiang Province, Harbin Veterinary Research Institute, Chinese Academy of Agricultural Sciences 111613 , Harbin, China; University of Michigan Medical School, Ann Arbor, Michigan, USA

**Keywords:** *Nidovirales*, arterivirus, EAV, MAVS, RIG-I, MARCH5, helicases, Nsp10, E3 ubiquitin ligase, Smurf1

## Abstract

Equine arteritis virus (EAV) is a positive-sense, single-stranded RNA virus that belongs to the family *Arteriviridae*, which also includes porcine reproductive and respiratory syndrome virus (PRRSV). EAV is the causative agent of equine viral arteritis (EVA), an economically important systemic, reproductive, and respiratory disease of equids. EAV infection triggers host innate immunity, yet the precise strategies employed by the virus to evade immune defenses and achieve productive infection are poorly characterized. In this study, it was observed that EAV infection can induce a cellular IFN response; however, EAV simultaneously significantly suppresses the expression of IFN-β in host cells. Our findings indicate that host cells sense EAV and elicit an IFN response via mitochondrial antiviral-signaling protein (MAVS)–mediated signal, but the virus can dampen this signaling to enhance infection. Further investigation showed that EAV nsp10 protein could interact with MAVS and promote its degradation. By screening, we found that the E3 ubiquitin ligases Smurf1 and MARCH5 are recruited by nsp10 to polyubiquitinate and degrade MAVS. Interestingly, the degradation of MAVS promoted by nsp10 depends on the dimerization of nsp10, which occurs through the interactions of zinc finger motifs. The CARD or PRR domain of MAVS and the 1A domain of nsp10 are responsible for the interaction between MAVS and nsp10. Moreover, we have identified the key amino acid residues that mediate the interactions between nsp10 and its binding partners. Specifically, D249, S287, and the S1/F39/N41 sites are critical for its binding to MAVS, Smurf1, and MARCH5, respectively. This study demonstrated a novel role for the arteriviral RNA helicase nsp10 in targeting MAVS to counteract innate immunity and reveals the mechanism by which EAV antagonizes MAVS.

**IMPORTANCE:**

Due to the MAVS functions as a “switch” in the immune signal transduction against RNA viruses, MAVS has emerged as the central regulatory target by viruses. Recently, researchers show increasing interest in viral evasion strategies targeting MAVS. The method of antagonism of MAVS by EAV is still unknown. To date, the roles of arteriviral RNA helicases, such as the EAV helicase nsp10, in regulating host cellular responses have received little research attention. In this study, we found that EAV nsp10 could mediate MAVS degradation through the proteasome via the E3 ubiquitin ligases Smurf1 and MARCH5. This is the first time that an arteriviral RNA helicase has been found to have an antagonistic effect on the innate immunity signaling pathway. Overall, our study reveals a novel mechanism by which EAV can evade host innate immunity and provides insight into potential therapeutic strategies for the control of arterivirus infection.

## INTRODUCTION

Equine arteritis virus (EAV) is an enveloped, positive-sense, single-stranded RNA virus that belongs to the family *Arteriviridae*, order *Nidovirales* (1, 2). This order includes coronaviruses, such as severe acute respiratory syndrome coronavirus type 2 (SARS-CoV-2) and Middle East respiratory syndrome (MERS)-CoV, known for their ability to cause severe respiratory infections in humans, as well as other significant veterinary coronaviruses affecting avian, porcine, and bovine species (3). Additionally, this order also comprises arteriviruses, including porcine reproductive and respiratory syndrome virus (PRRSV), which has seriously affected the pig industry (4). EAV is the etiological agent of equine viral arteritis (EVA), a disease affecting equids. EAV infection can be either asymptomatic or associated with a wide range of clinical signs (influenza-like syndromes), including pyrexia, depression, edema (ventral trunk, limbs, or scrotum), conjunctivitis, nasal discharge, and leukopenia (5–7). Stallions are susceptible to EAV infection, and semen from persistently infected stallions can infect and subsequently cause abortion in pregnant mares (7–9). EAV infection of pregnant mares can also result in the birth of congenitally infected foals that develop rapidly progressive and ultimately fatal bronchointerstitial pneumonia or pneumoenteric syndrome (10). EAV is an important respiratory and reproductive disease virus of equids, with worldwide distribution and causing significant economic losses to the horse industry. Moreover, EAV has broad species cell tropism and replicates *in vitro* in a variety of primary cell cultures and in a number of continuous cell lines, highlighting the potential risk of its transmission across host species barriers (11–14). These properties also make EAV an ideal RNA virus model for virus-cell interaction research.

The innate immune response constitutes the primary host defense mechanism against viral incursions. The nidoviruses are recognized by retinoic acid-inducible gene I (RIG-I) or melanoma differentiation-associated gene 5 (MDA5) (15, 16). After recognition of the virus, both RIG-I and MDA5 interact with the downstream adaptor protein MAVS (mitochondrial antiviral-signaling protein, also known as IPS1, VISA, and CARDIF) (17). MAVS plays a critical role in innate immunity. MAVS proteins on the mitochondrial outer membrane rapidly assemble into prion-like aggregates upon activation by RIG-I or MDA5 (18). Subsequently, MAVS triggers the activation of the cytosolic kinases IKK and TBK1, leading to the activation of transcription factors NF-κB and IRF3. These factors then translocate to the nucleus to stimulate the expression of type-I interferon (IFN-I) and other antiviral genes (17, 18). Most nidoviruses have developed strategies to antagonize the innate immune response by targeting MAVS in the IFN-I signaling pathway. For example, SARS-CoV-2 ORF10 can degrade MAVS through the autophagy pathway (19), and the 3C-like protease of PRRSV impedes IFN-β induction by cleaving MAVS (20). However, until now, it remains unclear whether EAV can antagonize the innate immune pathway of host cells by targeting MAVS.

Helicases are ATP-driven motor proteins that traverse their nucleic acid substrates and are capable of unwinding double-stranded regions. This can include protein displacement and the nucleation of larger RNA-protein complexes (21, 22). The nidoviruses also encode the helicases (23), including nsp10 in arteriviruses and nsp13 in coronaviruses (24). RNA helicases, such as EAV nsp10, have multiple functions—including replication of viral RNA, transcription of subgenomic mRNA (sg-mRNA), and virion biogenesis—and their sequence conservation across viral strains underscores their significance in the viral life cycle (24). In addition, the RNA helicases of coronaviruses have been shown to effectively suppress the production and signaling of IFN-I. Previous research has indicated that the nsp13 of SARS-CoV-2 can interact with the host kinase TBK1 and the transcription factor IRF3, potentially inhibiting IFN induction (25, 26). However, to date, the roles of arterivirus RNA helicases in regulating host cellular responses have received little research attention.

In this study, we found that EAV’s RNA helicase nsp10 mediates MAVS degradation through the proteasome via the E3 ubiquitin ligases Smurf1 and MARCH5. Our findings reveal an important mechanism of EAV evasion of the host antiviral immune response and demonstrate a novel role of arteriviral RNA helicases in antagonizing retinoic acid-inducible gene I-like receptor (RLR) signaling pathways.

## RESULTS

### EAV infection decreases MAVS expression to attenuate IFN-β levels

**Fig 1:**
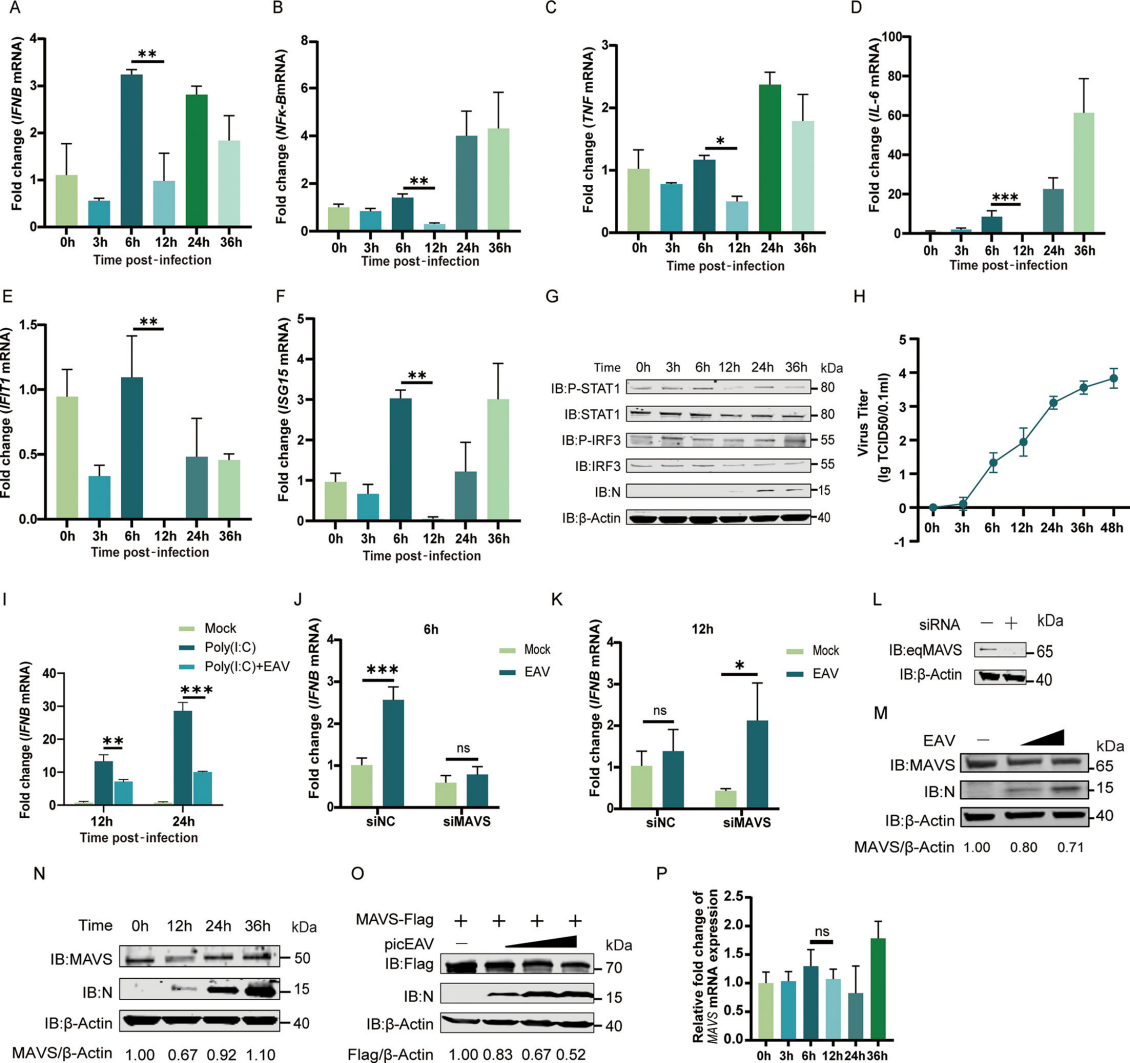
EAV infection downregulated MAVS expression to inhibit activation of IFN-β. (**A–F**) eMDMs were mock-treated or infected with EAV at an MOI of 1. Cells were harvested at 0, 3, 6, 12, 24, and 36 hpi, and levels of *IFNB* (**A**), *NF-κB* (**B**), *TNF* (**C**), *IL-6* (**D**), *IFIT1* (**E**), or *ISG15* (**F**) mRNA were visualized using qRT-PCR. The data represent the means ± SEM from three independent experiments. (**G**) eMDMs were mock-treated or infected with EAV at an MOI of 1. Cells were harvested at 0, 3, 6, 12, 24, and 36 hpi and subjected to immunoblot analysis with anti-pSTAT1, anti-STAT1, anti-pIRF3, anti-IRF3, anti-N, or anti-β-actin antibody. (**H**) eMDMs were mock-treated or infected with EAV at an MOI of 1. The supernatants were sampled at 0, 3, 6, 12, 24, 36, and 48 hpi, and the virus titers were determined using endpoint titration in RK13 cells. The data represent the means ± SEM from three independent experiments. (**I**) NBL-6 cells were mock-treated or treated with poly(I:C) (5 μg/mL) for 12 h and then mock-treated or infected with EAV at an MOI of 1. Cells were harvested at 12 and 24 hpi. The *IFNB* mRNA level was assessed using qRT-PCR. The data represent the means ± SEM from three independent experiments. (**J–L**) eMDMs cells were transfected with either scrambled siRNA or specific siRNA targeting equine MAVS (eqMAVS). At 24 h after transfection, cells were infected with EAV at an MOI of 1 (**J and K**) or subjected to immunoblot analysis with anti-eqMAVS or anti-β-actin antibody (**L**). Cells were harvested at 6 (**J**) and 12 (**K**) hpi, and *IFNB* mRNA levels were assessed using qRT-PCR. The data represent the means ± SEM from three independent experiments. (**M**) eMDMs cells were mock-treated or infected with EAV at an MOI of 0.5 or 1. Cells were harvested at 24 hpi and subjected to immunoblot analysis with anti-eqMAVS, anti-N, or anti-β-actin antibody. The results of the densitometry analysis to quantify the ratio of eqMAVS to β-actin are shown at the bottom (lane 1 set as 1). (**N**) eMDMs cells were mock-treated or infected with EAV at an MOI of 1. Cells were harvested at 0, 12, 24, and 36 hpi and subjected to immunoblot analysis with anti-eqMAVS, anti-N, or anti-β-actin antibody. The results of the densitometry analysis to quantify the ratio of eqMAVS to β-actin are shown at the bottom (lane 1 set as 1). (**O**) HEK293T was transfected with Flag-tagged MAVS and the picEAV infectious clone plasmid. Cells were harvested at 24 hpt and subjected to immunoblot analysis with anti-Flag, anti-N, or anti-β-actin antibody. The results of the densitometry analysis to quantify the ratio of Flag to β-actin are shown at the bottom (lane 1 set as 1). (**P**) eMDMs were mock-treated or infected with EAV at an MOI of 1. Cells were harvested at 0, 3, 6, 12, 24, and 36 hpi, and levels of *eqMAVS* mRNA were visualized using qRT-PCR. The data represent the means ± SEM from three independent experiments. (**A–P**) Significant differences between the different groups were determined using Student’s *t*-tests. NS, not significant, *P* > 0.05; *, *P* < 0.05; **, *P* < 0.01; ***, *P* < 0.001; ****, *P* < 0.0001.

Innate immunity is the first line of host defense against RNA virus infection. To characterize the host immune and inflammatory responses following EAV infection, target host cells (equine monocyte-derived macrophages [eMDMs]) were infected with EAV and then harvested at different times, and gene expression was analyzed. Our results showed that EAV infection induces the initial upregulation of *IFNB*, *NF-κB*, *TNF,* and *IL-6* at 6 h post-infection (Fig. 1A through D). Meanwhile, EAV also significantly upregulated the expression of the interferon-stimulated genes (ISGs) *IFIT1* and *ISG15* (Fig. 1E and F) and induced the phosphorylation of STAT1 and IRF3 (Fig. 1G).

The viruses have evolved various strategies to circumvent host antiviral defenses. Indeed, following early EAV infection, the levels of *IFNB*, *NF-κB*, *TNF*, *IL-6*, *IFIT1*, and *ISG15* all declined by 12 h post-infection compared to 6 h (Fig. 1A through F), with a concurrent decrease in the phosphorylation of STAT1 and IRF3 (Fig. 1G). The levels of these cytokines increased between 24 and 36 h post-infection and correlated with the rising viral titer (Fig. 1A through H). To explore whether EAV infection suppresses the innate immune response, we profiled the expression of *IFNB* in response to poly(I:C) stimulation followed by EAV infection. The results indicated that EAV inhibited the expression of *IFNB* in horse dermal fibroblast cells (NBL-6) (Fig. 1I). Consistent with our results, EAV has previously been reported to inhibit the induction of IFN-I to evade the immune response (27).

The RIG-I/MDA5 signaling pathway is crucial for triggering the induction of IFN-β in response to infections with diverse RNA viruses (28). In the RLR signaling pathway, MAVS is the only adaptor molecule that relays innate immune activation downstream of RIG-I or MDA5 (16). To investigate whether EAV targets equine MAVS to inhibit the RLR pathways, we designed small interfering RNAs (siRNAs) targeting MAVS and tested its role in EAV-triggered IFN-β inhibition. Our findings indicate that EAV could trigger the IFN-β pathway via a MAVS-mediated signal (Fig. 1J). In addition, EAV infection can suppress IFN-β expression via MAVS in eMDMs (Fig. 1K). The effects of MAVS knockdown have been shown in Fig. 1L. We have thus demonstrated EAV antagonism of MAVS. Immunoblotting experiments indicated that EAV inhibits MAVS expression in a multiplicity of infection (MOI)-dependent manner (Fig. 1M). At 12 h post-EAV infection, significant degradation of MAVS was observed in the cells. However, as the infection progressed and viral titers increased, MAVS expression gradually increased (Fig. 1N). To further verify that EAV can reduce MAVS expression, the ic-EAV infectious clone plasmid picEAV and MAVS-Flag were co-transfected into HEK293T cells. As expected, the expression of picEAV could reduce MAVS expression (Fig. 1O). EAV infection inhibits MAVS expression without significantly reducing its transcription (Fig. 1P). These data suggest that EAV can suppress the induction of IFN-β by reducing the MAVS expression in the RLR signaling pathway.

### EAV nsp10 protein reduces MAVS expression levels

**Fig 2:**
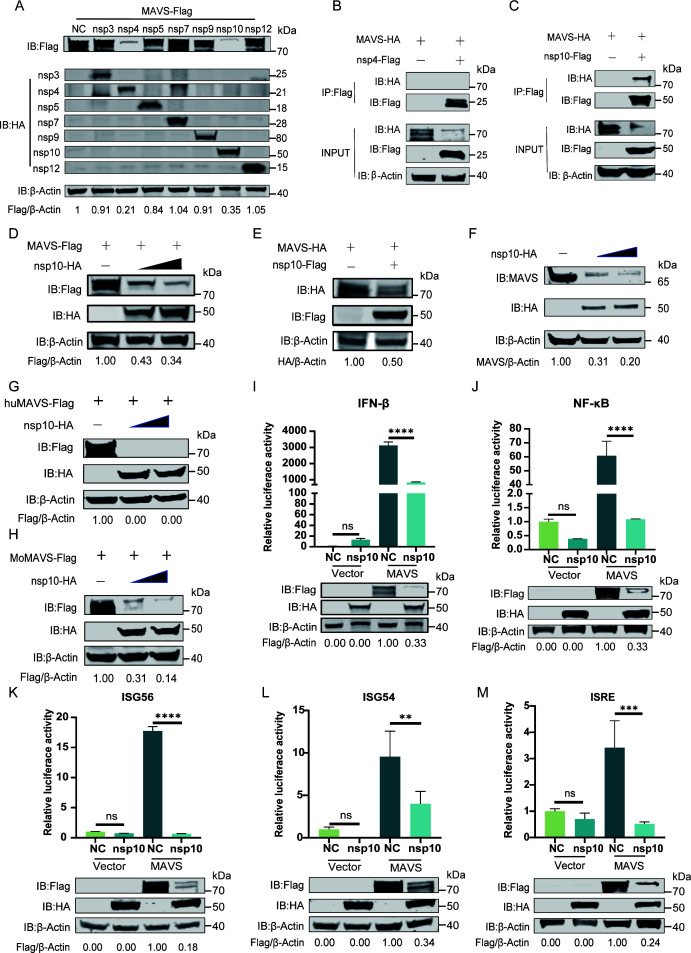
EAV nsp10 protein triggers MAVS degradation. (**A**) HEK293T cells were co-transfected with either empty vector or HA-tagged nsp3, nsp4, nsp5, nsp7, nsp9, nsp10, or nsp12 and Flag-tagged MAVS. Cells were harvested at 24 hpt and were assessed using immunoblot analysis with anti-Flag, anti-HA, or anti-β-actin antibody. The results of the densitometry analysis to quantify the ratio of Flag to β-actin are shown at the bottom (lane 1 set as 1). (**B and C**) HEK293T cells were co-transfected with MAVS-HA and either empty vector or nsp4-Flag (**B**) or nsp10-Flag (**C**). At 24 hpt, cells were harvested, co-immunoprecipitated with anti-Flag antibody, and further subjected to immunoblot analysis with anti-HA or anti-Flag antibody. Protein expression levels were subjected to immunoblot analysis of the lysates using anti-HA, anti-Flag, or anti-β-actin antibody. (**D**) HEK293T cells were transfected with either empty vector or nsp10-HA (1 or 2 μg/μL; wedge) and MAVS-Flag. Cells were harvested at 24 hpt and subjected to immunoblot analysis with anti-Flag, anti-HA, or anti-β-actin antibody. The results of the densitometry analysis to quantify the ratio of Flag to β-actin are shown at the bottom (lane 1 set as 1). (**E**) HEK293T cells were transfected with either empty vector or nsp10-Flag and MAVS-HA. Cells were harvested at 24 hpt and subjected to immunoblot analysis with anti-Flag, anti-HA, or anti-β-actin antibody. The results of the densitometry analysis to quantify the ratio of Flag to β-actin are shown at the bottom (lane 1 set as 1). (**F**) NBL-6 cells were transfected with either empty vector or nsp10-HA (1 or 2 μg/μL; wedge). Cells were harvested at 24 hpt and subjected to immunoblot analysis with anti-eqMAVS, anti-HA, or anti-β-actin antibody. The results of the densitometry analysis to quantify the ratio of eqMAVS to β-actin are shown at the bottom (lane 1 set as 1). (**G and H**) HEK293T cells were transfected with either empty vector or nsp10-HA (1 or 2 μg/μL; wedge) and human MAVS (huMAVS)-Flag (**G**) or monkey MAVS (moMAVS)-Flag (**H**). Cells were harvested at 24 hpt and subjected to immunoblot analysis with anti-Flag, anti-HA, or anti-β-actin antibody. The results of the densitometry analysis to quantify the ratio of Flag to β-actin are shown at the bottom (lane 1 set as 1). (**I–M**) HEK293T cells were co-transfected with a plasmid expressing nsp10 (or an empty vector control), the pRL-TK Renilla luciferase transfection control, and one of the following firefly luciferase reporter plasmids: pGL3-IFN-β-Luc (**I**), NF-κB-Luc (**J**), ISG56-Luc (**K**), ISG54-Luc (**L**), or ISRE-Luc (**M**). Simultaneously, cells were mock-treated or transfected with MAVS-Flag plasmid. After 24 h, the cells were collected, and then cell lysates were analyzed for IFN-β-Luc (**I**), NF-κB-Luc (**J**), ISG56-Luc (**K**), ISG54-Luc (**L**), or ISRE-Luc (**M**) activity. Expression levels of the proteins were analyzed by immunoblot analysis of the lysates with anti-Flag, anti-HA, or anti-β-actin antibody. The results of the densitometry analysis to quantify the ratio of Flag to β-actin are shown at the bottom (lane 3 set as 1). Significant differences between the different groups were determined using Student’s *t*-tests. NS, not significant, *P* > 0.05; *, *P* < 0.05; **, *P* < 0.01; ***, *P* < 0.001; ****, *P* < 0.0001. The data represent the means ± SEM from three independent experiments.

Previous studies have shown that some non-structural proteins (NSPs) of EAV play key roles in suppressing IFN production (27, 29). Since EAV infection inhibited the expression of MAVS, we next screened a panel of viral NSPs that suppress the expression of MAVS protein. HEK293T cells were co-transfected with plasmids expressing EAV’s NSPs and MAVS. An empty vector was used as a negative control. As shown in Fig. 2A, two viral NSPs, nsp4 and nsp10, impaired the expression of MAVS protein. To explore whether these two NSPs interacted with MAVS, we performed co-immunoprecipitation (Co-IP) assays. Interestingly, the results showed that there is no direct interaction between nsp4 and MAVS (Fig. 2B); however, nsp10 was found to interact with MAVS (Fig. 2C). Therefore, in this study, we selected nsp10 protein for further investigation.

To verify downregulation of MAVS expression by nsp10, HEK293T cells co-transfected with MAVS-Flag and different concentrations of nsp10-HA were analyzed using immunoblotting. We found that nsp10 reduced MAVS expression level in a dose-dependent manner (Fig. 2D). Moreover, a reverse immunoblotting experiment was performed with MAVS-HA and nsp10-Flag, and the results also confirmed that the nsp10 can downregulate MAVS expression (Fig. 2E). We then assessed the expression levels of endogenous MAVS in NBL-6 cells and found that nsp10 overexpression also reduced endogenous MAVS expression in a dose-dependent manner (Fig. 2F). Certain domains and functions of MAVS are conserved between species, so we investigated whether EAV nsp10 can reduce MAVS expression in other species. We therefore investigated the effect of nsp10 on MAVS from different species. Interestingly, we found that EAV nsp10 markedly reduced expression of human and monkey MAVS (Fig. 2G and H). Next, to explore the impact of nsp10-induced MAVS suppression on the innate immune process, we then evaluated IFN-β promoter activity with MAVS overexpression. HEK293T cells were co-transfected with MAVS and plasmids encoding the EAV nsp10 protein or an empty vector as a control. As expected, this assay demonstrated that EAV nsp10 protein can inhibit IFN-β promoter activity induced by MAVS overexpression, compared with the empty vector control (Fig. 2I). Consistent with the above result, nsp10 significantly suppresses the promoter activity of other innate immune molecules—including NF-κB, ISG56, ISG54, and ISRE—induced by MAVS overexpression (Fig. 2J through M). Taken together, these data suggest that EAV nsp10 protein is a general negative regulator of the MAVS-dependent antiviral immune and inflammatory responses.

### EAV nsp10 protein interacts with MAVS in the cytoplasm

**Fig 3:**
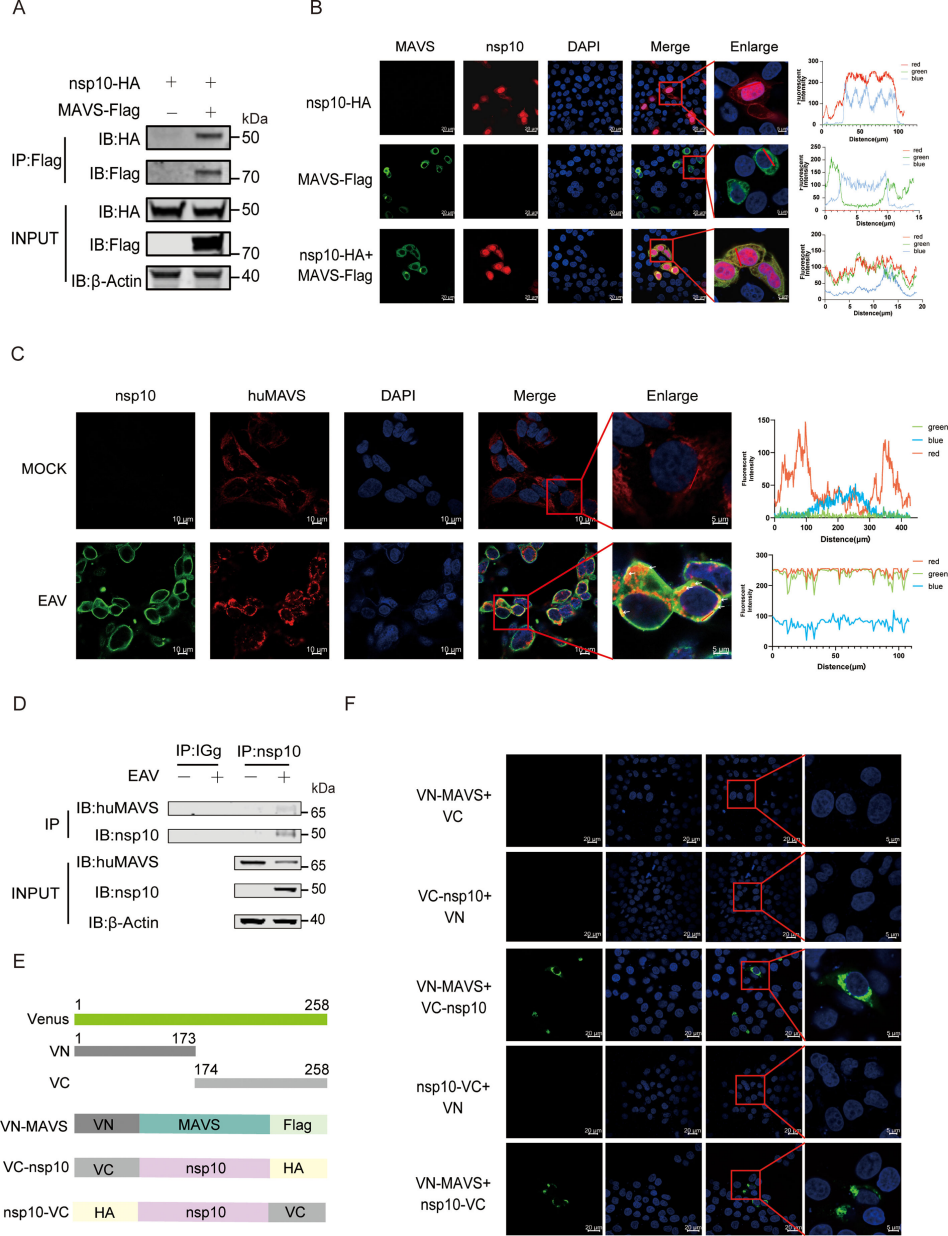
EAV nsp10 interacts with MAVS in the cytoplasm. (**A**) HEK293T cells were co-transfected with nsp10-HA and either empty vector or MAVS-Flag. At 24 hpt, cells were harvested, co-immunoprecipitated with anti-Flag antibody, and subjected to immunoblot analysis with anti-HA or anti-Flag antibody. Protein expression levels were assessed using immunoblot analysis of the lysates with anti-HA, anti-Flag, or anti-β-actin antibody. (**B**) HeLa cells were individually transfected with either nsp10-HA or MAVS-Flag and co-transfected with nsp10-HA and MAVS-Flag. At 24 hpt, cells were harvested and subjected to immunofluorescence assay with anti-Flag or anti-HA antibody (scale bar, 20 μm; 5 μm). Shown is an intensity profile of the linear region of interest (ROI) across the HeLa cell co-stained with nsp10 and MAVS. (**C**) HEK293 cells were mock-treated or infected with EAV at an MOI of 1. At 24 hpi, cells were harvested and subjected to immunofluorescence assay with anti-nsp10 or anti-MAVS antibody (scale bar, 20 μm; 5 μm). Shown is an intensity profile of the linear region of interest (ROI) across the HEK293 cell co-stained with nsp10 and MAVS. (**D**) HEK293 cells were mock-treated or infected with EAV at an MOI of 1. At 24 hpt, cells were harvested, co-immunoprecipitated with anti-nsp10 antibody, and subjected to immunoblot analysis with anti-huMAVS or anti-nsp10 antibody. Protein expression levels were assessed using immunoblot analysis of the lysates with anti-huMAVS, anti-nsp10, or anti-β-actin antibody. (**E**) Schematic of the BiFC fusion proteins. (**F**) HeLa cells were co-transfected with VN-MAVS and VC, VC-nsp10 and VN, VC-nsp10 and VN-MAVS, nsp10-VC and VN, or nsp10-VC and VN-MAVS. At 24 hpt, BiFC green fluorescent signals together with the expression of nsp10 and MAVS expression were visualized using confocal microscopy (scale bar, 20 μm; 5 μm).

To investigate the interaction between nsp10 and MAVS, a reverse Co-IP experiment was performed using nsp10-HA and MAVS-Flag, in which we found that nsp10 interacts with MAVS (Fig. 3A). Previous studies have demonstrated that the nsp10 protein of the PRRSV is localized in either the nucleus or the cytoplasm (30). We next sought to determine the cellular location of EAV’s nsp10-MAVS interaction. Confocal microscopy revealed that MAVS and nsp10 co-localized in the cytoplasm (Fig. 3B). To further investigate whether EAV nsp10 associates with endogenous MAVS during the course of infection, cells were infected with EAV and harvested for analysis. Nsp10 was predominantly localized in the cytoplasm, and colocalization with MAVS was also observed in infected cells (Fig. 3C). The endogenous Co-IP results also demonstrated an interaction between MAVS and nsp10 during EAV infection (Fig. 3D). To confirm the specificity and subcellular distribution of the nsp10-MAVS interaction, a bimolecular fluorescence complementation (BiFC) assay that could directly visualize protein-protein interactions in living cells was performed. Three constructs were generated: VN-MAVS, in which MAVS-Flag was fused with the N-terminal residues 1 to 173 of Venus (VN); VC-nsp10, in which the N-terminal of EAV nsp10-HA was fused with C-terminal residues 174 to 238 of Venus (VC); and nsp10-VC, in which the C-terminal of EAV nsp10-HA was fused with VC (Fig. 3E). We visualized the BiFC signals using confocal microscopy. We detected an interaction between nsp10 (VC-nsp10 or nsp10-VC) and MAVS, with strong subcellular localization in the cytoplasm (Fig. 3F), which was consistent with the results of our confocal microscopy experiments. Collectively, these data demonstrate that EAV nsp10 can interact with MAVS in the cytoplasm.

### EAV nsp10 protein promotes MAVS degradation through the ubiquitin-proteasome pathway

**Fig 4:**
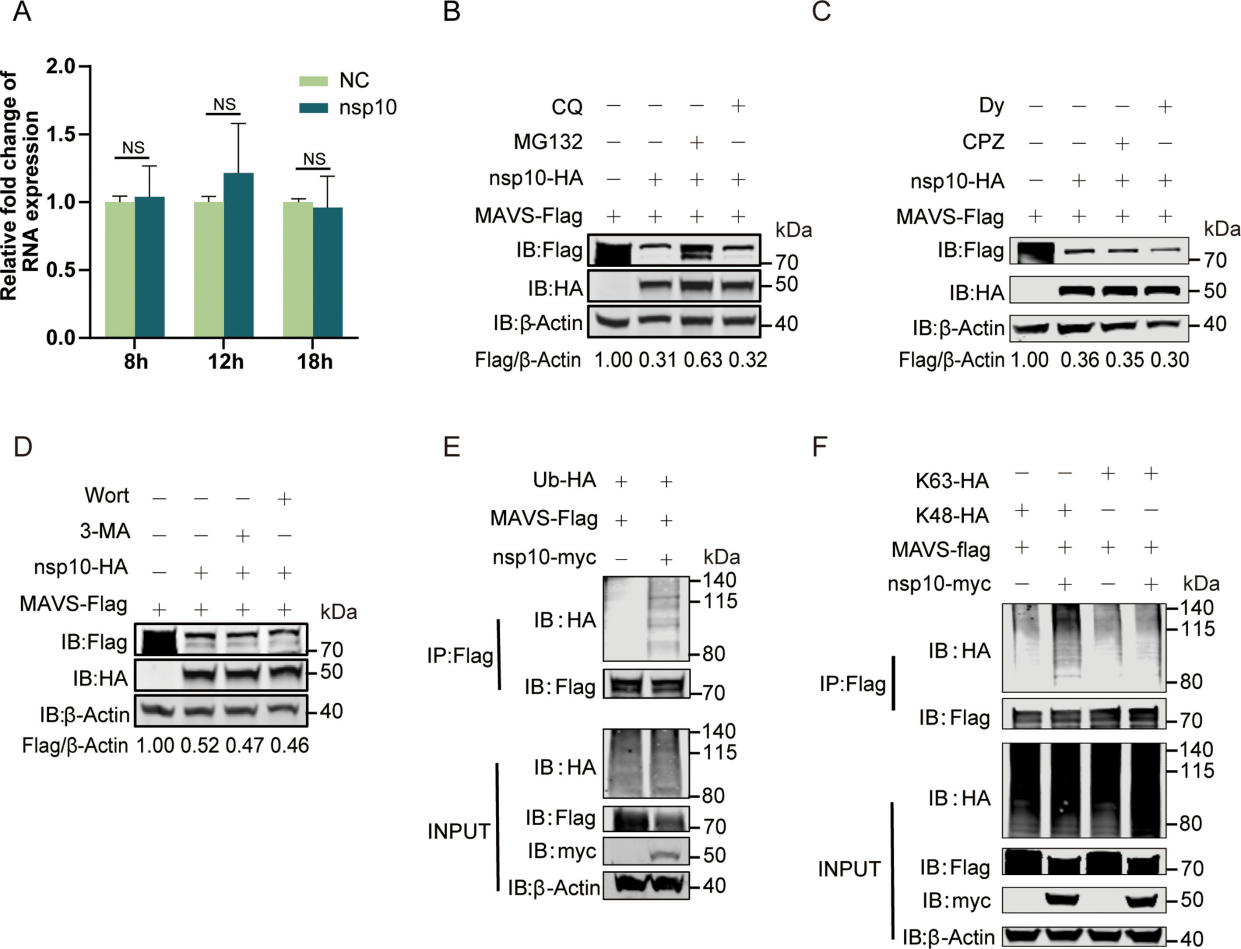
EAV nsp10 protein triggers MAVS degradation through the ubiquitin-proteasome pathway. (**A**) HEK293T cells were co-transfected with nsp10-HA and either empty vector or MAVS-Flag. Cells were harvested at 8, 12, and 18 hpt, and MAVS mRNA levels were assessed using qPCR. Significant differences between the different groups were determined using Student’s *t*-tests. NS, not significant, *P* > 0.05; *, *P* < 0.05; **, *P* < 0.01; ***, *P* < 0.001; ****, *P* < 0.0001. The data represent the means ± SEM from three independent experiments. (**B–D**) HEK293T cells were co-transfected with nsp10-HA and either empty vector or MAVS-Flag and maintained in the presence or absence of the proteasome inhibitor MG132 (20 μM) or lysosome inhibitor chloroquine (CQ; 20 μM) for 8 h (**B**). For endocytosis inhibitors, the transfected cells were treated with dynasore (Dy; 20 μM) and chlorpromazine (CPZ; 20 μM) for 8 h prior to immunoblot analysis (**C**). For the autophagy inhibitor, the transfected cells were treated with 3-MA (5 mM) and wortmannin (Wort; 25 nM) for 8 h prior to immunoblot analysis (**D**). Cells were harvested and subjected to immunoblot analysis with anti-HA, anti-Flag, or anti-β-actin antibody. The results of the densitometry analysis to quantify the ratio of Flag to β-actin are shown at the bottom (lane 1 set as 1). (**E**) HEK293T cells were co-transfected with MAVS-Flag, HA-ubiquitin (Ub), and either empty vector or nsp10-myc were maintained in the presence of the proteasome inhibitor MG132 (20 μM, 8 h prior to immunoprecipitation). At 24 hpt, cells were harvested, co-immunoprecipitated with anti-Flag antibody, and subjected to immunoblot analysis with anti-HA, anti-Flag, anti-myc, or anti-β-actin antibody. (**F**) HEK-293T cells were co-transfected with MAVS-Flag, HA-ubiquitin (K48), or HA-ubiquitin (K63), and either empty vector or nsp10-myc, and maintained in the presence of the proteasome inhibitor MG132 (20 μM, 8 h prior to immunoprecipitation). At 24 hpt, cells were harvested, co-immunoprecipitated with anti-Flag antibody, and subjected to immunoblot analysis with anti-HA, anti-Flag, anti-myc, or anti-β-actin antibody.

EAV nsp10 protein can dramatically inhibit the expression of MAVS; however, EAV nsp10 had no effect on MAVS transcription (Fig. 4A). Therefore, we speculated that EAV nsp10 reduces MAVS protein expression via a degradation pathway. The ubiquitin-proteasome and autophagy-lysosome pathways are major systems that control protein degradation in eukaryotic cells. To determine which of these pathways is required for nsp10-mediated MAVS degradation, we investigated protein degradation in nsp10- and MAVS-co-transfected cells with or without the proteasome inhibitor MG132; the lysosome inhibitor chloroquine (CQ) (Fig. 4B); the endocytosis inhibitors dynasore (Dy) and chlorpromazine (CPZ) (Fig. 4C); or the autophagy inhibitors 3-methyladenine (3-MA) and wortmannin (Wort) (Fig. 4D). Remarkably, MG132 greatly restored MAVS protein in the presence of nsp10, whereas the lysosome, endocytosis, and autophagy inhibitors had no apparent effect on MAVS degradation.

Proteasomes remove proteins that are tagged with ubiquitin via proteolytic cleavage. Thus, we next analyzed the polyubiquitination of MAVS in the presence or absence of nsp10 expression. Using Co-IP, we confirmed that nsp10 protein increased the ubiquitination of MAVS (Fig. 4E). Ubiquitin can be added to target proteins, such as MAVS, via distinct lysine residues of ubiquitin. Two main lysine residues, K48 and K63, are best characterized for their distinct functions in mammalian cells. To determine which ubiquitin residue was important in nsp10-mediated MAVS degradation, we overexpressed K48-HA and K63-HA ubiquitin mutants and examined subsequent MAVS ubiquitination. We found that ubiquitination of nsp10-mediated MAVS was undertaken by ubiquitin-K48 but not by ubiquitin-K63 (Fig. 4F). Collectively, these results demonstrate that nsp10 can promote MAVS degradation via the K48-linked ubiquitin-proteasome system.

### The E3 ubiquitin ligases MARCH5 and Smurf1 are involved in EAV nsp10 protein-induced MAVS degradation

**Fig 5:**
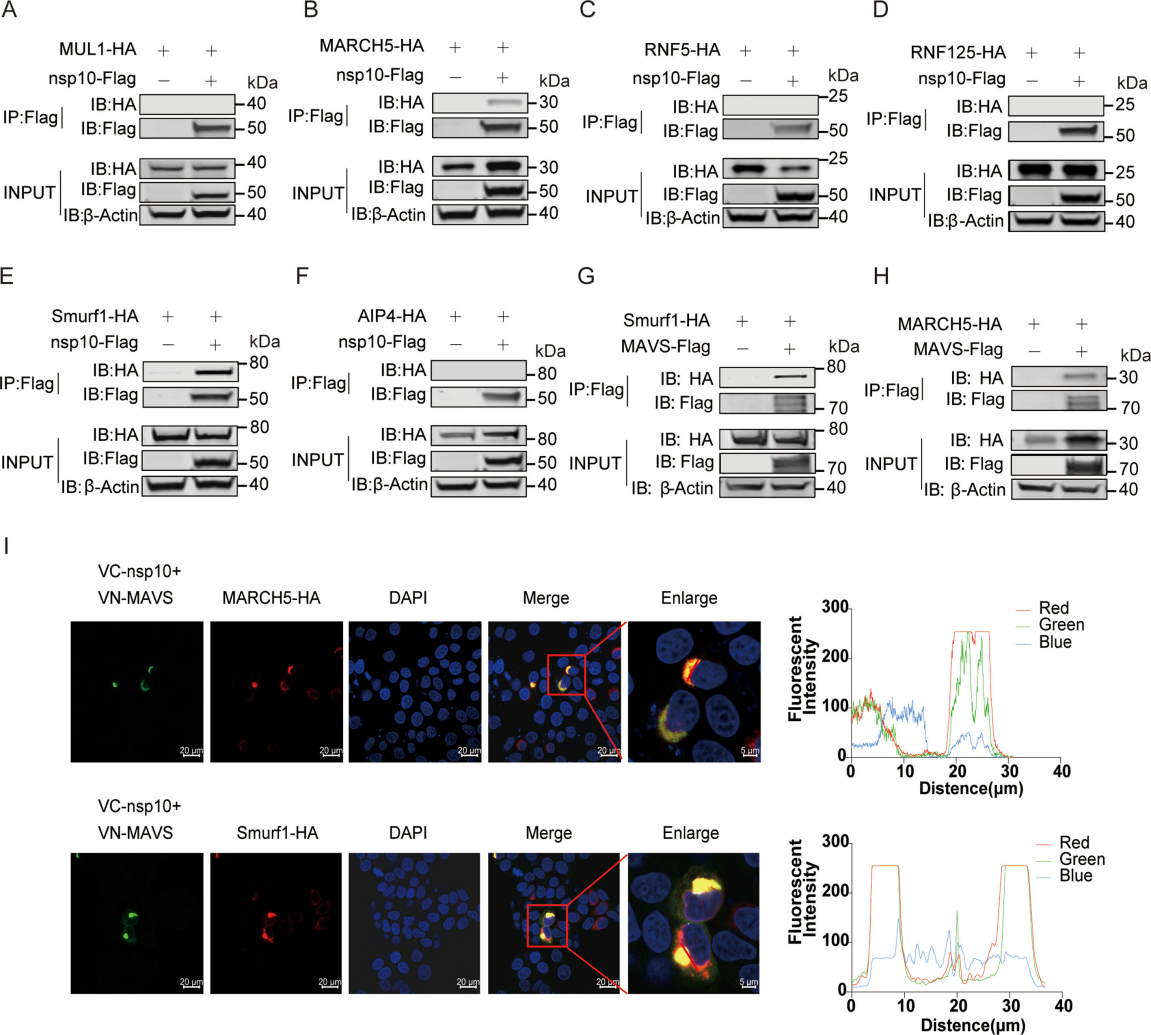
Characterization of Smurf1 and MARCH5 as the E3 ubiquitin ligases for nsp10-mediated MAVS degradation. (**A–F**) HEK293T cells were co-transfected with nsp10-Flag or the empty vector and HA-tagged MUL1 (**A**), MARCH5 (**B**), RNF5 (**C**), RNF125 (**D**), Smurf1 (**E**), or AIP4 (**F**). At 24 hpt, cells were harvested, co-immunoprecipitated with anti-Flag antibody, and further subjected to immunoblot analysis with anti-HA or anti-Flag antibodies. Protein expression levels of the proteins were assessed using immunoblot analysis of the lysates with anti-HA, anti-Flag, or anti-β-actin antibody. (**G and H**) HEK293T cells were co-transfected with MAVS-Flag or the empty vector and HA-tagged Smurf1 (**G**) or MARCH5 (**H**). At 24 hpt, cells were harvested, co-immunoprecipitated with anti-Flag antibody, and subjected to immunoblot analysis with anti-HA or anti-Flag antibody. Protein expression levels were assessed using immunoblot analysis of the lysates with anti-HA, anti-Flag, or anti-β-actin antibody. (**I**) Investigation of MAVS, nsp10, and Smurf1 or MARCH5 interaction using BiFC assays. HeLa cells were co-transfected with VC-nsp10, VN-MAVS, and Smurf1-HA or MARCH5-HA. Smurf1 or MARCH5 protein was stained with rabbit anti-HA antibody followed by Alexa Fluor 555-conjugated rabbit anti-mouse antibody. BiFC green fluorescent signals together with the expression of Smurf1 or MARCH5 were visualized using confocal microscopy (scale bar, 20 μm; 5 μm).

K48-linked ubiquitylation mediates the degradation of MAVS and acts as an important negative regulator of innate antiviral immunity. The RLR-mediated innate immune response is critically regulated by the ubiquitin modification system, with multiple E3 ubiquitin ligases—including RNF5, MARCH5, AIP4, MUL1, Smurf1, and RNF125—playing pivotal roles in its fine-tuning (31–36). To investigate which E3 ubiquitin ligase is involved in EAV nsp10-mediated MAVS degradation, we investigated the interactions between the E3 ubiquitin ligases and EAV nsp10. Our Co-IP results indicated that nsp10 interacted with MARCH5 and Smurf1, but not with RNF5, AIP4, MUL1, or RNF125 (Fig. 5A through F). In addition, the results also demonstrated that Smurf1 and MARCH5 separately specifically interacted with MAVS (Fig. 5G and H). To further confirm the interactions between nsp10, MAVS, and Smurf1 or MARCH5, two E3 ligases were separately co-transfected with VN-MAVS and VC-nsp10 into HeLa cells. The two E3 ligases both completely co-localized with the MAVS-nsp10 BiFC complex (Fig. 5I).

**Fig 6:**
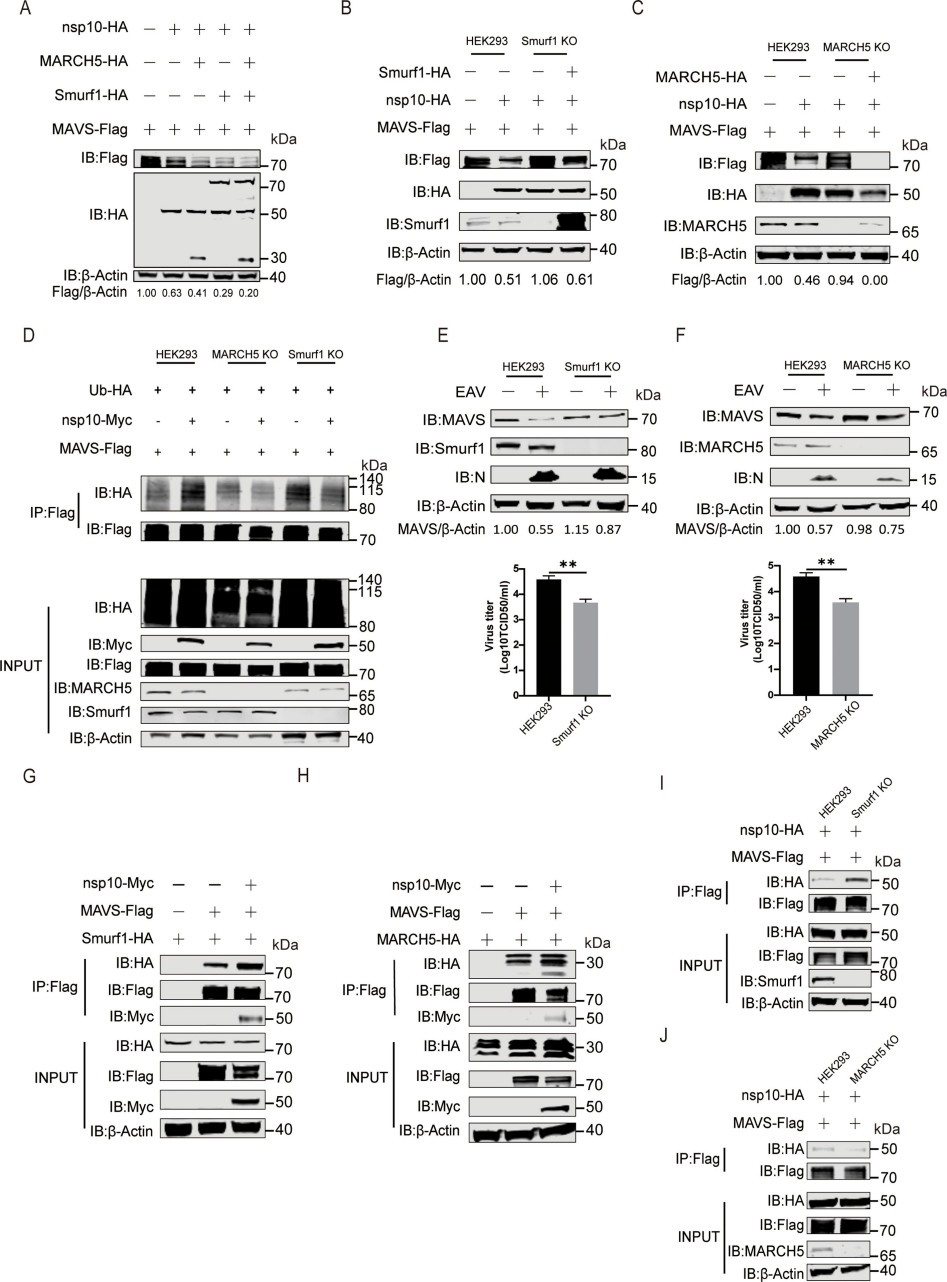
Smurf1 and MARCH5 synergistically regulate MAVS degradation during EAV infection. (**A**) HEK293T cells were co-transfected with MAVS-Flag, nsp10-HA, and HA-tagged MARCH5, Smurf1, or MARCH5 and Smurf1. At 24 hpt, cells were harvested and subjected to immunoblot analysis with anti-HA, anti-Flag, or anti-β-actin antibody. The results of the densitometry analysis to quantify the ratio of Flag to β-actin are shown at the bottom (lane 1 set as 1). (**B**) HEK293 cells and Smurf1 KO cells were co-transfected with MAVS-Flag, nsp10-HA, and HA-tagged Smurf1 or empty vector. At 24 hpt, cells were harvested and subjected to immunoblot analysis with anti-HA, anti-Flag, or anti-β-actin antibody. The results of the densitometry analysis to quantify the ratio of Flag to β-actin are shown at the bottom (lane 1 set as 1). (**C**) HEK293 cells and MARCH5 KO cells were co-transfected with MAVS-Flag, nsp10-HA, and HA-tagged MARCH5 or empty vector. At 24 hpt, cells were harvested and subjected to immunoblot analysis with anti-HA, anti-Flag, or anti-β-actin antibody. The results of the densitometry analysis to quantify the ratio of Flag to β-actin are shown at the bottom (lane 1 set as 1). (**D**) HEK293 cells, Smurf1 KO cells, and MARCH5 KO cells were co-transfected with MAVS-Flag, HA-ubiquitin (Ub), and either empty vector or nsp10-myc and maintained in the presence of the proteasome inhibitor MG132 (20 μM, 8 h prior to immunoprecipitation). At 24 hpt, cells were harvested, co-immunoprecipitated with anti-Flag antibody, and subjected to immunoblot analysis with anti-HA, anti-Flag, anti-myc, or anti-β-actin antibody. (**E and F**) HEK293 cells and Smurf1 KO cells (**E**) or MARCH5 KO cells (**F**) were infected with EAV at the MOI of 1. At 24 hpi, cells were harvested and subjected to immunoblot analysis with anti-huMAVS, anti-N, anti-Smurf1 (**E**), anti-MARCH5 (**F**), or anti-β-actin antibody. The results of the densitometry analysis to quantify the ratio of huMAVS to β-actin are shown at the bottom (lane 1 set as 1). The supernatants were sampled at 0 and 24 h post-infection, and the virus titers were determined using endpoint titration in RK13 cells. Significant differences between the different groups were determined using Student’s *t*-tests. NS, not significant, *P* > 0.05; *, *P* < 0.05; **, *P* < 0.01; ***, *P* < 0.001; ****, *P* < 0.0001. The data represent the means ± SEM from three independent experiments. (**G and H**) HEK293T cells were co-transfected with HA-tagged Smurf1 (**G**) or MARCH5 (**H**), MAVS-Flag, and nsp10-myc or empty vector. At 24 hpt, cells were harvested, co-immunoprecipitated with anti-Flag antibody, and subjected to immunoblot analysis with anti-HA, anti-Flag, anti-myc, or anti-β-actin antibody. (**I and J**) HEK293 cells and Smurf1 KO cells or MARCH5 KO cells were co-transfected with MAVS-Flag and nsp10-HA. At 24 hpt, cells were harvested, co-immunoprecipitated with anti-Flag antibody, and subjected to immunoblot analysis with anti-HA, anti-Flag, anti-myc, or anti-β-actin antibody.

To determine the role of the two E3 ligases in MAVS degradation by nsp10, MARCH5 or Smurf1 was overexpressed alone or together with MAVS and nsp10 in HEK293T cells. The results showed that both co-expression and individual overexpression of MARCH5 and Smurf1 significantly enhance nsp10-mediated degradation of MAVS (Fig. 6A). We next designed single guide RNAs (sgRNAs) targeting the E3 ligases, MARCH5 or Smurf1, and constructed MARCH5 or Smurf1 knockout (KO) HEK293 cells using a CRISPR/Cas9 system. By using MARCH5 or Smurf1 KO cells, we tested their roles in EAV nsp10-mediated MAVS degradation. The results indicated that the depletion of MARCH5 or Smurf1 effectively restores MAVS levels degraded by nsp10, whereas re-expression of MARCH5 or Smurf1 leads to a reduction in MAVS expression (Fig. 6B and C). Moreover, we performed the experiment to detect ubiquitination blot for MAVS in MARCH5 or Smurf1 KO cells. The results demonstrated that, compared with WT cells, the ubiquitination level of MAVS was reduced following nsp10 transfection in either MARCH5 or Smurf1 KO cells (Fig. 6D). These data suggest that the two E3 ubiquitin ligases, MARCH5 and Smurf1, were involved in the ubiquitination and degradation of MAVS by EAV nsp10. To further verify the role of MARCH5 or Smurf1 in EAV replication, we measured viral TCID50 and MAVS expression levels in MARCH5 or Smurf1 KO cells at 24 h post-infection. The results demonstrated that the deficiency of either MARCH5 or Smurf1 led to a partial restoration of MAVS expression relative to WT cells, which was associated with reduced EAV replication efficiency (Fig. 6E and F). Next, we want to know how the interaction of nsp10 with MAVS and the two E3 ligases leads to MAVS degradation. To assess the interactions, HEK293T cells were co-transfected with HA-Smurf1 (Fig. 6G) or HA-MARCH5 (Fig. 6H), MAVS-Flag, and nsp10-myc (or empty vector). The Co-IP results indicated that nsp10 could enhance the interaction between MAVS and MARCH5 or Smurf1 (Fig. 6G and H). Meanwhile, we also validated the alterations in the interaction between nsp10 and MAVS in MARCH5 or Smurf1 KO cells. Interestingly, the results showed that Smurf1 knockout enhanced the association between MAVS and nsp10, whereas MARCH5 knockout reduced the likelihood of MAVS-nsp10 complex formation (Fig. 6I and J). This may be attributed to differences in the interaction domains among nsp10, MAVS, and MARCH5 or Smurf1, as well as potential competitive binding. Therefore, we need to systematically examine their interaction domains.

### Domain map of nsp10 and MAVS interaction

**Fig 7:**
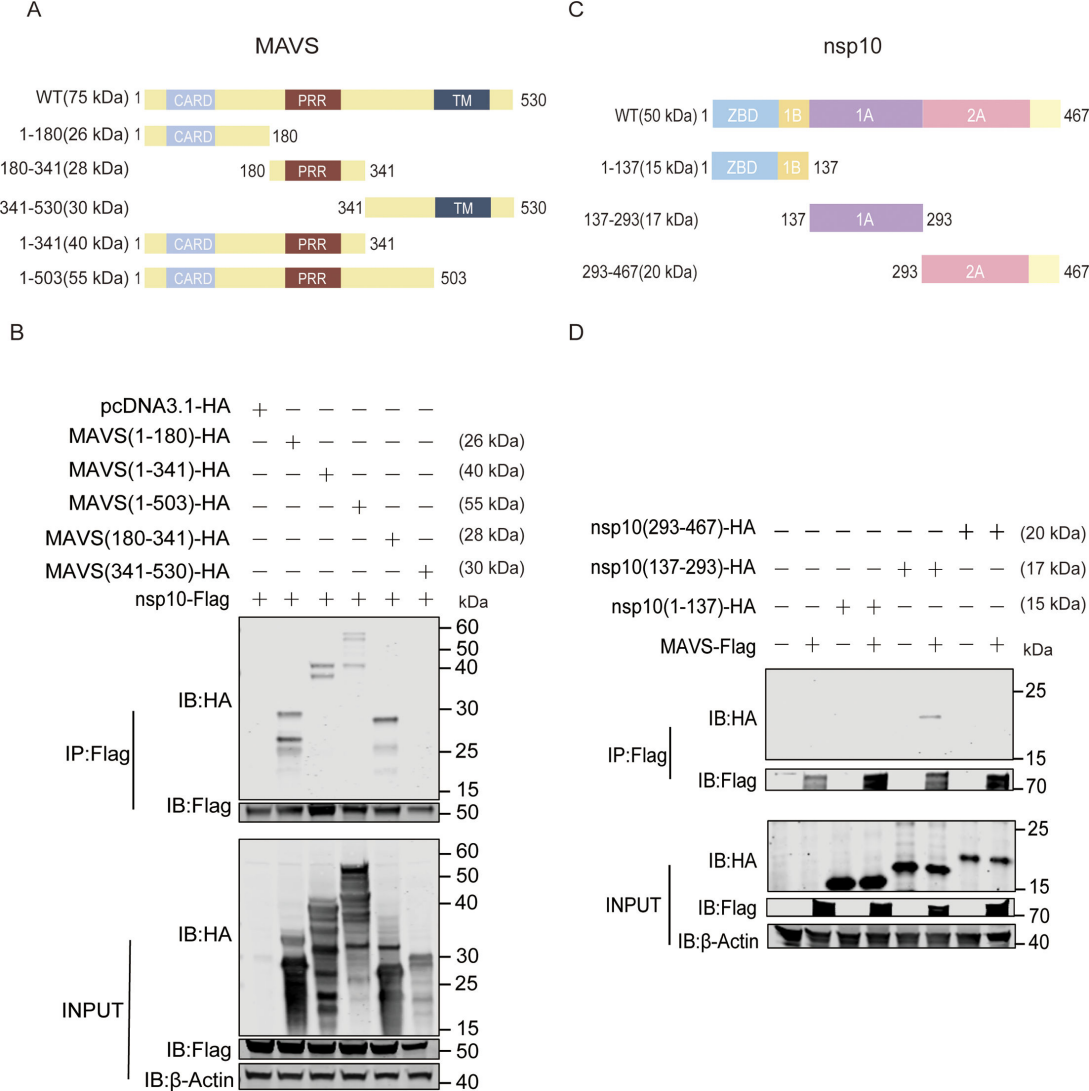
Domain mapping of the nsp10-MAVS interaction. (**A**) Schematics of a series of HA-tagged truncated MAVS constructs. (**B**) HEK293T cells were co-transfected with nsp10-Flag and either an empty vector or aa 1–180, aa 1–341, aa 1–503, aa 180–341, or aa 341–530 truncated HA-MAVS and maintained in the presence of the proteasome inhibitor MG132 (20 μM, 8 h prior to immunoprecipitation). At 24 hpt, cells were harvested, co-immunoprecipitated with anti-Flag antibody, and subjected to immunoblot analysis with anti-HA or anti-Flag antibody. Protein expression levels were assessed using immunoblot analysis of the lysates with anti-HA, anti-Flag, or anti-β-actin antibody. (**C**) Schematics of a series of HA-tagged truncated nsp10 constructs. (**D**) HEK293T cells were co-transfected with MAVS-Flag and either empty vector or aa 1–137, aa 137–293, or aa 293–467 truncated nsp10-HA and maintained in the presence of the proteasome inhibitor MG132 (20 μM, 8 h prior to immunoprecipitation). At 24 hpt, cells were harvested, co-immunoprecipitated with anti-Flag antibody, and subjected to immunoblot analysis with anti-HA or anti-Flag antibody. Protein expression levels were assessed using immunoblot analysis of the lysates with anti-HA, anti-Flag, or anti-β-actin antibody.

EAV nsp10 degrades MAVS by interacting with it. To identify which domains are required for the nsp10-MAVS interaction, we performed Co-IP analysis to determine the domains responsible for this interaction. As shown in Fig. 7A, MAVS contains an N-terminal caspase activation and recruitment domain (CARD)-like domain, a middle proline-rich region (PRR) domain, and a C-terminal transmembrane (TM) domain. To analyze the critical domain of MAVS responsible for nsp10-MAVS interaction, nsp10-Flag was co-transfected with either empty vector, WT MAVS-HA, and truncated HA-MAVS (aa 1–180, aa 180–341, aa 341–530, aa 1–341, or aa 1– 503), and then analyzed with Co-IP. The results showed that either the CARD or PRR domain of MAVS was required for its interaction with nsp10 (Fig. 7B). The EAV nsp10 is the most conserved replicase subunit of the viral RNA synthesis machinery. It contains an N-terminal zinc-binding domain (ZBD), the two RecA-like domains 1A and 2A of the helicase core (HEL1), and an additional regulatory domain 1B, a short linker that connects HEL1 domain with ZBD domain (Fig. 7C). Different mutations of nsp10 plasmids were constructed and were co-transfected with MAVS into HEK293T cells. The Co-IP results showed that only domain 1A of nsp10 was responsible for the interaction with MAVS (Fig. 7D). These results suggest that the EAV nsp10 1A domain and the CARD and PRR domains of MAVS are critical for the interaction between nsp10 and MAVS.

### Domain map of the interactions nsp10-Smurf1 and nsp10-MARCH5

**Fig 8:**
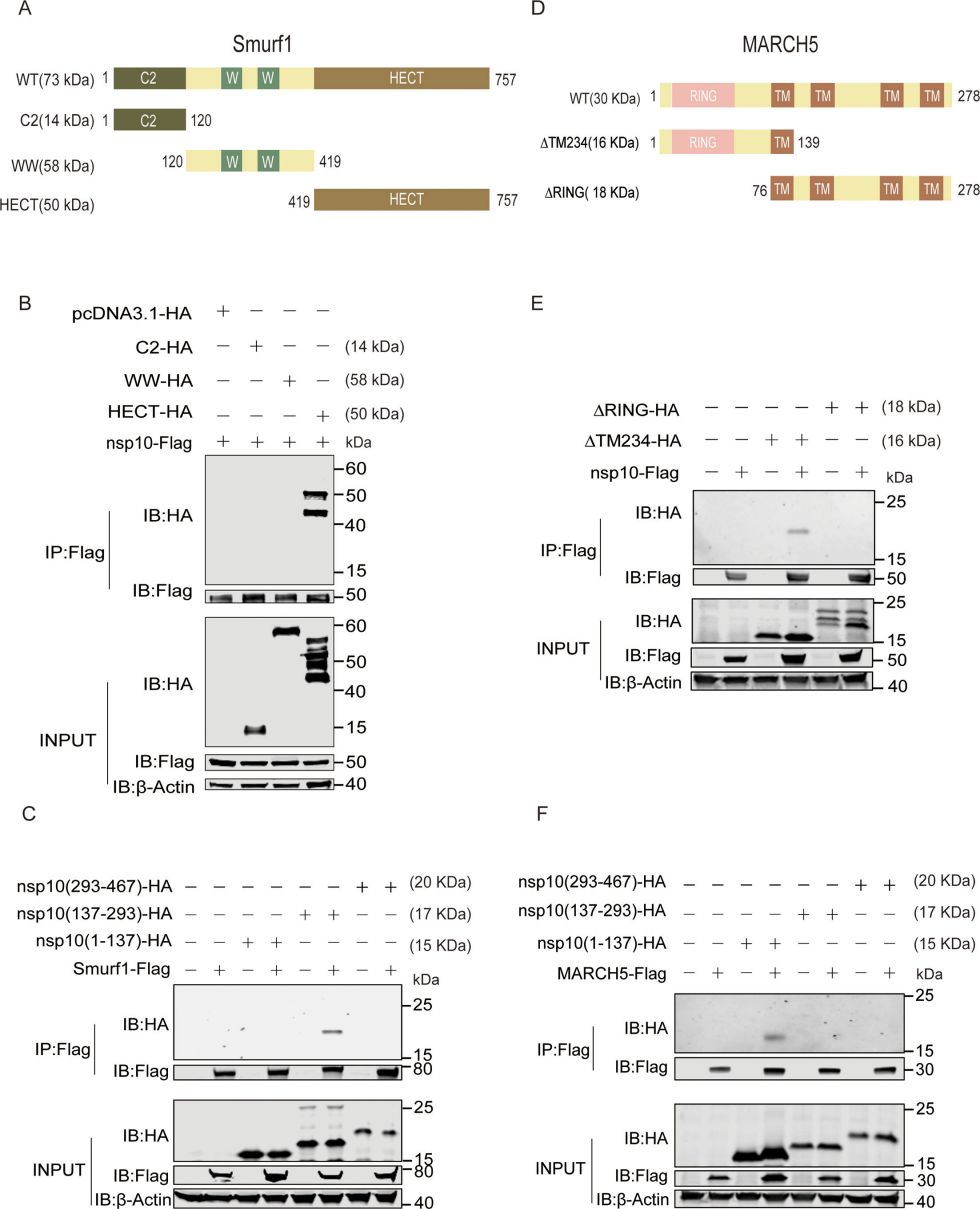
Domain mapping of the nsp10-Smurf1 and nsp10-MARCH5 interactions. (**A**) Schematics of a series of HA-tagged truncated Smurf1 constructs. (**B**) HEK293T cells were co-transfected with nsp10-Flag and either empty vector or aa 1–120, aa 120–419, or aa 419–757 truncated HA-Smurf1. At 24 hpt, cells were harvested, co-immunoprecipitated with anti-Flag antibody, and subjected to immunoblot analysis with anti-HA or anti-Flag antibody. Protein expression levels were assessed using immunoblot analysis of the lysates with anti-HA, anti-Flag, or anti-β-actin antibody. (**C**) HEK293T cells were co-transfected with Smurf1-Flag and either empty vector or aa 1–137, aa 137–293, or aa 293–467 truncated nsp10-HA. At 24 hpt, cells were harvested, co-immunoprecipitated with anti-Flag antibody, and subjected to immunoblot analysis with anti-HA or anti-Flag antibody. Protein expression levels were assessed using immunoblot analysis of the lysates with anti-HA, anti-Flag, or anti-β-actin antibody. (**D**) Schematics of a series of HA-tagged truncated MARCH5 constructs. (**E**) HEK293T cells were co-transfected with nsp10-Flag and either empty vector or aa 1–139 or aa 76–278 truncated HA-MARCH5. At 24 hpt, cells were harvested, co-immunoprecipitated with anti-Flag antibody, and subjected to immunoblot analysis with anti-HA or anti-Flag antibody. Protein expression levels were assessed using immunoblot analysis of the lysates with anti-HA, anti-Flag, or anti-β-actin antibody. (**F**) HEK293T cells were co-transfected with MARCH5-Flag and either empty vector or aa 1–137, aa 137–293, or aa 293–467 truncated nsp10-HA. At 24 hpt, cells were harvested, co-immunoprecipitated with anti-Flag antibody, and further subjected to immunoblot analysis with anti-HA or anti-Flag antibody. Protein expression levels were assessed using immunoblot analysis of the lysates with anti-HA, anti-Flag, or anti-β-actin antibody.

EAV nsp10 recruits two E3 ubiquitin ligases, MARCH5 and Smurf1, to promote MAVS degradation. Smurf1 belongs to the HECT (homologous to E6AP C-terminus) E3s family. Structurally, Smurf1 contains four key domains: a catalytic HECT domain in the C-terminus, two WW domains (WW1 and WW2), and a phospholipid binding C2 domain in the N-terminus region (37). To map the domains of Smurf1 that are critical for the interaction with nsp10, we generated three truncated Smurf1 mutants: C2 (aa 1–120), WW (aa 120–419), and HECT (aa 419–757) (Fig. 8A). Then, nsp10-Flag was co-transfected with either empty vector, WT Smurf1-HA, or one of the three truncated Smurf1-HA, and the results were analyzed using Co-IP. We found that the HECT domain of Smurf1 was sufficient for the interaction with nsp10 (Fig. 8B). Next, we wanted to know which domain of nsp10 was necessary for this process. The major functional domains of nsp10 are illustrated in Fig. 7C. Our Co-IP results demonstrated that the RecA-like domain 1A of nsp10, but not any of the other domains, was crucial for the interaction with Smurf1 (Fig. 8C). Collectively, these results demonstrated that the HECT domain of Smurf1 and the 1A domain of nsp10 are crucial for the Smurf1-nsp10 interaction.

MARCH5 is a mitochondrially localized RING-finger E3 ligase. It contains a characteristic ubiquitin ligase RING domain at the N-terminus and four TM α-helices at the C-terminus. To investigate the functional domain of MARCH5 responsible for the MARCH5-nsp10 interaction, we generated a series of truncation mutants of MARCH5 (Fig. 8D), as previously described (32). Our subsequent Co-IP analysis showed that nsp10 interacted with the region comprising the RING of MARCH5 (Fig. 8E). Next, to investigate which domain of nsp10 was responsible for the interaction with MARCH5, we constructed different mutations of nsp10 (Fig. 7C). In subsequent Co-IP assays, we observed that nsp10-ZBD, but not nsp10-1A or nsp10-2A, interacted with MARCH5 (Fig. 8F). Altogether, these data demonstrated that the RING domain of MARCH5 and the ZBD domain of nsp10 are crucial for the MARCH5-nsp10 interaction.

### The dimerization of nsp10 promotes the degradation of MAVS

**Fig 9:**
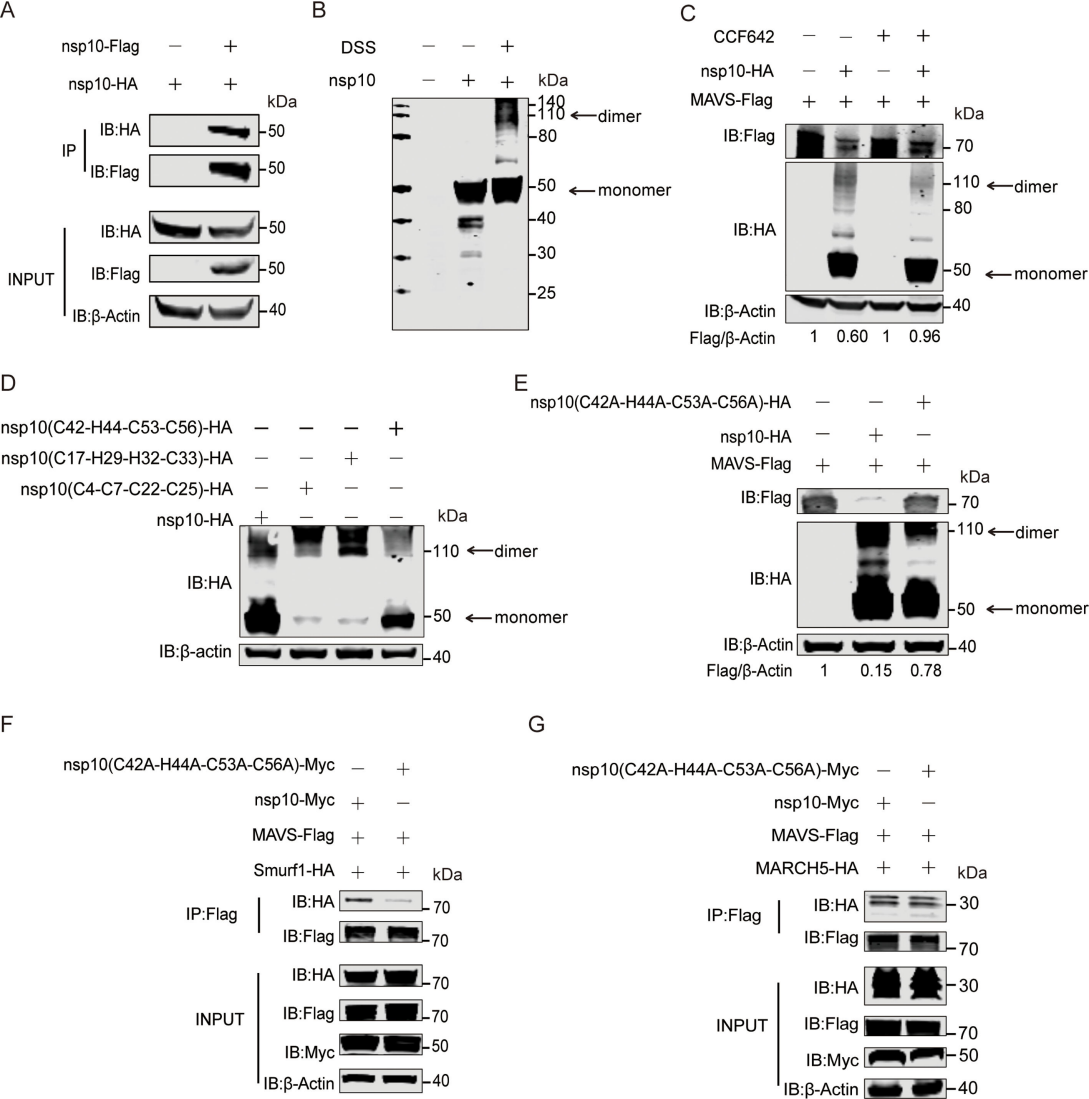
The nsp10-MAVS interaction is based on the dimerization of nsp10. (**A**) HEK293T cells were co-transfected with nsp10-Flag and HA-tagged nsp10. At 24 hpt, cells were harvested, co-immunoprecipitated with anti-Flag antibody, and subjected to immunoblot analysis with anti-HA or anti-Flag antibody. Protein expression levels were assessed using immunoblot analysis of the lysates with anti-HA, anti-Flag, or anti-β-actin antibody. (**B**) HEK293T cells were transfected with nsp10-HA. At 24 hpt, cells were harvested; cells were pretreated with 250 µM DSS cross-linker for 30 min before protein extraction and subjected to immunoblot analysis with anti-HA or anti-β-actin antibody. (**C**) HEK293T cells were co-transfected with nsp10-HA and either empty vector or MAVS-Flag and maintained in the presence or absence of the PDI inhibitor CCF642 (3 μM) for 8 h. The cells were harvested; cells were pretreated with 250 µM DSS cross-linker for 30 min before protein extraction and then subjected to immunoblot analysis with anti-Flag, anti-HA, or anti-β-actin antibody. The results of the densitometry analysis to quantify the ratio of Flag to β-actin are shown at the bottom (lane 1 and 3 set as 1). (**D**) HEK293T cells were transfected with nsp10-HA, nsp10 (C4A-C7A-C22A-C25A)-HA, nsp10 (C17A-H29A-H32A-C33A)-HA, or nsp10 (C42A-H44A-C53A-C56A)-HA. At 24 hpt, cells were harvested, pretreated with 250 µM DSS cross-linker for 30 min before protein extraction, and subjected to immunoblot analysis with anti-HA or anti-β-actin antibody. The results of the densitometry analysis to quantify the ratio of HA to β-actin are shown at the bottom (lane 1 set as 1). (**E**) HEK293T cells were co-transfected with MAVS-Flag and nsp10-HA or the nsp10 (C42A-H44A-C53A-C56A)-HA. At 24 hpt, cells were harvested and pretreated with 250 µM DSS cross-linker for 30 min before protein extraction. Cells were then subjected to immunoblot analysis with anti-Flag, anti-HA, or anti-β-actin antibody. The results of the densitometry analysis to quantify the ratio of Flag to β-actin are shown at the bottom (lane 1 set as 1). (**F and G**) HEK293T cells were co-transfected with HA-tagged Smurf1 (**F**) or MARCH5 (**G**), MAVS-Flag, and either nsp10-myc or the nsp10 (C42A-H44A-C53A-C56A)-myc. At 24 hpt, cells were harvested, co-immunoprecipitated with anti-Flag antibody, and subjected to immunoblot analysis with anti-HA, anti-Flag, anti-myc, or anti-β-actin antibody.

The main structure of ZBD is composed of two adjacent zinc finger motifs with distinct architectures, along with a treble-clef zinc finger motif (24). Previous research has indicated that some of the zinc finger motifs form a stable dimerization complex (38, 39). Therefore, we investigated whether nsp10 exhibits dimerization and whether the dimerization of nsp10 could promote the degradation of MAVS. We constructed a Flag-tagged nsp10 protein expression plasmid, nsp10-Flag, and then co-transfected it together with nsp10-HA into HEK293T cells. Co-IP experiments showed that EAV nsp10 demonstrates self-interaction (Fig. 9A). To further confirm a dimerization of nsp10, we performed cross-linking experiments with the cross-linker, N-hydroxysuccinimidyl ester disuccinimidyl suberate (DSS). HEK293T cells were transfected with nsp10-HA, and then, the cross-linking was performed. In the presence of the cross-linker DSS, nsp10 was detected with an apparent molecular weight of 80 to 140 kDa, which indicated that nsp10 exhibits dimerization (Fig. 9B). To investigate whether the dimerization of nsp10 plays a role in the degradation of MAVS, CCF642, which specifically inhibits the reductase activity of the family of protein disulfide isomerases (PDI), was used to decrease the dimerization of nsp10. The results demonstrated a restoration of MAVS protein levels following the reduction in nsp10 dimerization (Fig. 9C). The three zinc ions of nsp10 are coordinated by distinct sets of residues: the first by Cys4, Cys7, Cys22, and Cys25; the second by Cys17, His29, His32, and Cys33; and the third by Cys42, His44, Cys53, and Cys56 (24). We next introduced mutations in the residues coordinating the three zinc ions. A significant reduction in nsp10 dimerization occurred following the mutation of the coordinating residues of the third zinc ion (Fig. 9D). Then, we co-transfected the nsp10 variant carrying mutated residues for the coordination of the third zinc ion together with MAVS into HEK293T cells and found the MAVS protein was greatly restored in the presence of this mutant (Fig. 9E). To investigate the mechanism by which nsp10 dimerization influences MAVS degradation, we assessed the ability of a monomeric nsp10 mutant to form complexes with MAVS and the E3 ubiquitin ligases Smurf1 and MARCH5. Co-IP results demonstrated that the nsp10 monomeric mutant significantly impaired the interaction between MAVS and Smurf1, while the association between MAVS and MARCH5 remained unaffected (Fig. 9F and G). These data together demonstrate that the dimerization of nsp10 plays an important role in the degradation of MAVS.

### Key sites of interactions between nsp10 and MAVS, MARCH5, or Smurf1

**Fig 10:**
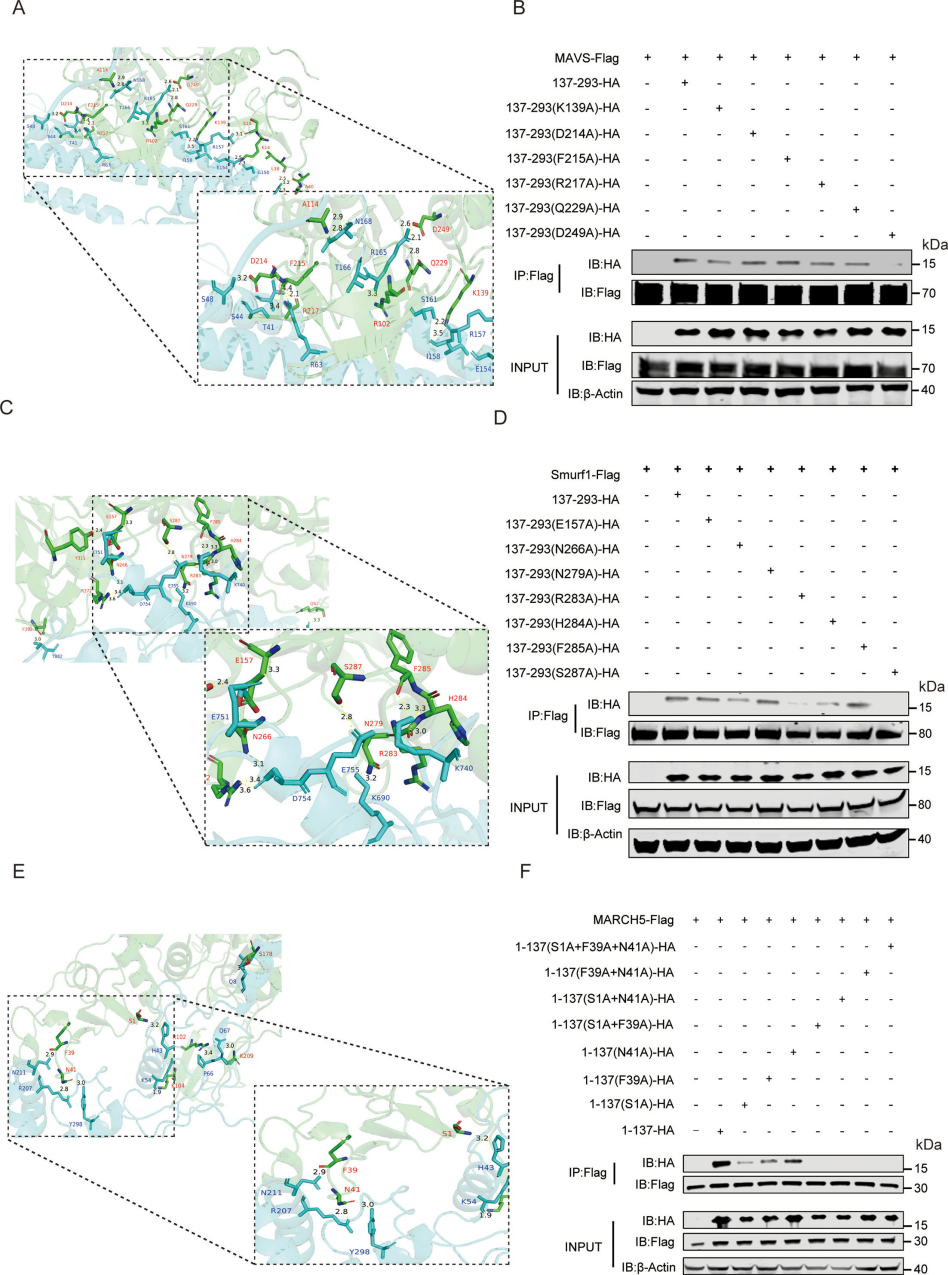
Analysis of key amino acid sites in the MAVS-nsp10-Smurf1 and MAVS-nsp10-MARCH5 interactions. (**A**) Structure-based prediction of the interface between nsp10 (green) and MAVS (blue). Crystal structure analysis of the nsp10-MAVS conjugate shows non-covalent interactions between nsp10 and MAVS (possible hydrogen bonds are indicated by yellow dashed lines, with distances in Å). K139, D214, F215, R217, Q229, and D249 of nsp10 facilitate the formation of covalent bonds. (**B**) EK293T cells were co-transfected with MAVS-Flag and either empty vector or nsp10-HA or the nsp10 (K139A)-HA (from Lys to Ala), nsp10 (D214A)-HA (from Asp to Ala), nsp10 (F215A)-HA (from Phe to Ala), nsp10 (R217A)-HA (from Arg to Ala), nsp10 (Q229A)-HA (from Gln to Ala), or nsp10 (D249A)-HA (from Asp to Ala). At 24 hpt, cells were harvested, co-immunoprecipitated with anti-Flag antibody, and subjected to immunoblot analysis with anti-HA or anti-Flag antibody. (**C**) Structure-based prediction of the interface between nsp10 (green) and Smurf1 (blue). Crystal structure analysis of the nsp10-Smurf1 conjugate shows non-covalent interactions between nsp10 and Smurf1 (possible hydrogen bonds are indicated by yellow dashed lines, with distances in Å). E157, N266, N279, R283, H284, F285, and S287 of nsp10 facilitate the formation of covalent bonds. (**D**) HEK293T cells were co-transfected with Smurf1-Flag and either empty vector or nsp10-HA or the nsp10 (E157A)-HA (from Glu to Ala), nsp10 (N266A)-HA (from Asn to Ala), nsp10 (N279A)-HA (from Asn to Ala), nsp10 (R283A)-HA (from Arg to Ala), nsp10 (H284A)-HA (from His to Ala), nsp10 (S287A)-HA (from Ser to Ala), or nsp10 (D249A)-HA (from Asp to Ala). At 24 hpt, cells were harvested, co-immunoprecipitated with anti-Flag antibody, and subjected to immunoblot analysis with anti-HA or anti-Flag antibody. (**E**) Structure-based prediction of the interface between nsp10 (green) and MARCH5 (blue). Crystal structure analysis of the nsp10-MARCH5 conjugate shows non-covalent interactions between nsp10 and MARCH5 (possible hydrogen bonds are indicated by yellow dashed lines, with distances in Å). S1, F39, and N41 of nsp10 facilitate the formation of covalent bonds. (**F**) HEK293T cells were co-transfected with MARCH5-Flag and either empty vector or nsp10-HA or nsp10 (S1A)-HA (from Ser to Ala), nsp10 (F39A)-HA (from Phe to Ala), nsp10 (N41A)-HA (from Asn to Ala), nsp10 (S1A + F39A)-HA (from Ser and Phe to Ala), nsp10 (S1A + N41A)-HA (from Ser and Asn to Ala), nsp10 (F39A + N41A)-HA (from Phe and Asn to Ala), or nsp10 (S1A + F39A + N41A)-HA (from Ser, Phe, and Asn to Ala). At 24 hpt, cells were harvested, co-immunoprecipitated with anti-Flag antibody, and subjected to immunoblot analysis with anti-HA or anti-Flag antibody.

EAV nsp10 interacts with MAVS and targets it for degradation. To investigate in detail the sites through which nsp10 interacts with MAVS, we constructed a MAVS-nsp10 interaction model by molecular simulation. We found that the residues K139, D214, F215, R217, Q229, and D249 of the critical nsp10 1A domain support the interaction with MAVS (Fig. 10A). These residues were then verified as being important in the nsp10-MAVS interaction through the introduction of mutations followed by Co-IP assays. As shown in Fig. 10B, the resulting mutant D249A of nsp10 showed reduced binding affinity to MAVS. We also investigated the interaction sites between nsp10 and the two E3 ubiquitin ligases Smurf1 and MARCH5. The molecular simulation results showed that the residues of E157, N266, N279, R283, H284, F285, and S287, or S1, F39, and N41 in the 1A or ZBD domains of nsp10 competitively bound to Smurf1 or MARCH5. The Co-IP experiments showed that only mutant S287A of nsp10 showed reduced binding affinity to Smurf1. Similarly, the mutants S1A, F39A, and N41A of nsp10 singly reduced nsp10 binding affinity to MARCH5, and mutations at any two of these sites simultaneously can eliminate the interaction of nsp10 with MARCH5 (Fig. 10C through F). In conclusion, the key site D249 has been identified as critical for the interaction between MAVS and nsp10. Our data also reveal that the key nsp10 sites S287 and S1/F39/N41 are responsible for the interaction of nsp10 with Smurf1 and MARCH5, respectively.

## DISCUSSION

**Fig 11:**
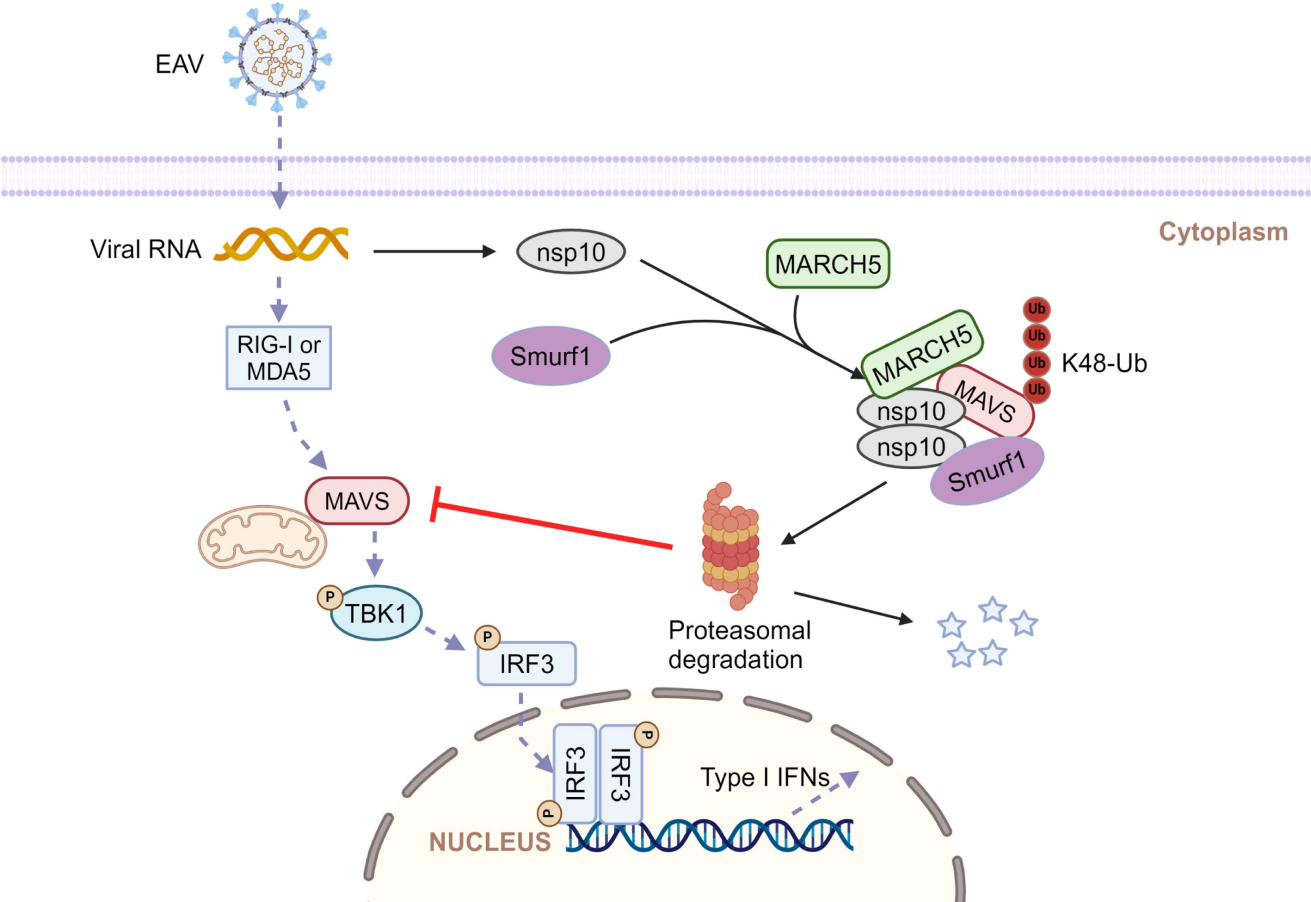
Schematic diagrams illustrating how EAV negatively regulates RLR pathway activation by degrading MAVS. Virus infection results in activation of the RLR pathway and production of type I IFN. To escape this host innate immunity, the EAV RNA helicase nsp10 recruits two E3 ubiquitin ligases, Smurf1 and MARCH5, to degrade MAVS. These ligases catalyze K48-linked polyubiquitination, leading to proteasome-dependent degradation of MAVS. These processes lead to the inhibition of the RLR signaling pathway and downregulation of IFN-β.

MAVS plays vital roles in antiviral signal transduction and immune homeostasis due to its function as a switch in immune signal transduction and is a central target for both virus and hosts (40). Emerging evidence suggests that specific viral proteins can subvert the host immune system by targeting the MAVS molecule, thereby dampening the innate immune signaling (41, 42). Many proteins of the nidoviruses, including SARS-CoV-2 NP, SARS-CoV-2 ORF10, PEDV 3CL^pro^, and PRRSV 3CL^pro^, have been reported to inhibit the key signaling adaptor MAVS, which is activated by RIG-I/MDA5, limiting the host innate immune defense response to the virus (19, 20, 43, 44). In this study, we demonstrated that EAV, which also belongs to the nidoviruses, can decrease MAVS levels and subsequently inhibit RLR-induced innate immune activation. Furthermore, our data showed that EAV nsp10 targets MAVS for degradation via the ubiquitin proteasome pathway. The mechanism involves the recruitment of the E3 ligases Smurf1 and MARCH5 by the EAV nsp10 protein via a stepwise biological process to mediate K48-linked polyubiquitination of MAVS (Fig. 11). Consequently, the MAVS-mediated innate signaling pathway is disrupted, leading to the suppression of the interferon responses. The current study thus revealed a novel strategy mediated by the nsp10 protein of EAV, in which the virus utilized the host proteasome system to degrade a critical molecule in the innate signaling pathway and block the IFN-I response.

However, RNA viruses, like SARS-CoV-2, induce host interferon (IFN) responses via multiple signaling pathways. These encompass not only the cytosolic RIG-I-like receptor (RLR) pathway but also endosomal Toll-like receptor (TLR) pathways. Both converge to activate the transcription factors IRF3 and IRF7, thereby driving the production of Type I and III IFNs. Furthermore, the cGAS-STING pathway—primarily a sensor for cytosolic DNA—can be secondarily activated by virus-induced cellular damage, further contributing to the IFN response (45). For example, the replication of EAV is greatly facilitated in cGAS-deficient cells (46). Similarly, PRRSV, another member of the Arteriviridae family, has also been reported to activate IFN through the cGAS pathway (47). Moreover, a dynamic shift in signaling predominance occurs among PRR pathways to coordinate antiviral immunity and control inflammation (48). Our results showed that IFN expression with siMAVS was higher than siNC in EAV-infected cells (Fig. 1K). The absence of MAVS does not completely abrogate IFN induction upon EAV infection, implying that the virus may activate host defenses through alternative routes.

We first showed that EAV infection induces MAVS protein degradation without affecting its mRNA levels. The nsp10 protein of EAV has been demonstrated to interact with MAVS and acts as a direct suppressor of MAVS (Fig. 2 and 3). EAV nsp10, as an RNA helicase, has helicase and adenosine triphosphatase (ATPase) activities, enabling the unwinding of dsRNAs and propelling processes, such as viral genome replication, transcription of subgenomic mRNA, assembly of viral particles, and synthesis of subgenomic RNA (sgRNA) (49, 50). Previous studies have shown that RNA helicases are essential for viral replication in the nidoviruses EAV (50) and the b-CoV murine hepatitis virus (51), and are presumed to be essential in all nidoviruses (52). To date, research into the RNA helicases of nidoviruses has focused on their central role in the replication and transcription of the viral genome. However, the potential functions of these helicases beyond the viral replication processes, particularly in innate immunity, remain largely elusive. To our knowledge, we describe here for the first time a novel function of nidoviral RNA helicases in the promotion of protein ubiquitination and degradation, which effectively inhibits the immune signaling pathways by degrading the key adaptor MAVS.

Unlike other arteriviruses, EAV can replicate *in vitro* in a large variety of cells, such as horse dermal fibroblast cells, Marc-145 cells derived from the embryonic kidney of African green monkeys, and human cervical cancer cells (HeLa) (53). In this study, we found that the nsp10 protein of EAV degrades not only equine MAVS (Fig. 2D) but also that from humans and monkeys (Fig. 2G and H). In order to replicate in cells from different species, after entering the cell, EAV needs to first break through the MAVS-mediated innate immune response in the host cell. Our data indicated that the EAV nsp10 protein can effectively reduce cellular levels of MAVS, so we speculated that EAV can antagonize innate immunity by targeting MAVS to allow it to replicate in cells from different species. This suggests that nsp10 might be a key factor in the adaptability of EAV to cells from different host species.

In this study, we found that not only the nsp10 protein but also the nsp4 protein can reduce expression levels of MAVS (Fig. 2A). However, we found no interaction between nsp4 and MAVS (Fig. 2B). Previous studies suggest that RNA viruses can alter the mitochondrial metabolism and homeostasis, ultimately leading to mitochondrial damage and blocking of the interferon response via MAVS (19, 54). Therefore, we speculated that the nsp4 protein of EAV might impair the mitochondria to decrease MAVS expression. The confocal results demonstrate that nsp4 localizes to mitochondria and induces mitochondrial fragmentation, suggesting that nsp4 downregulates MAVS expression through modulation of mitochondrial integrity. The precise mechanism remains to be elucidated in future studies (Fig. S1).

A series of E3 ubiquitin ligases have been reported to be involved in the process of ubiquitination and proteasomal degradation of MAVS (33, 55). Smurf1 has been identified as a potential E3 ubiquitin ligase involved in the degradation of MAVS after its self-ubiquitination (56). Previous research has reported that infection with RNA viruses promotes the interaction between Smurf1 and MAVS, which culminates in the ubiquitin-dependent degradation of MAVS (57). Lys7 and Lys500 of MAVS were shown to be polyubiquitinated by MARCH5, a mitochondrial membrane-bound E3 that effectively dissolves MAVS aggregates by specifically targeting them for degradation (32). Interestingly, in this study, we found that EAV nsp10 can recruit two E3 ubiquitin ligases, MARCH5 and Smurf1 (Fig. 5B and E), and to target MAVS for K48-linked ubiquitination (Fig. 4F). When MARCH5 and Smurf1 are co-expressed, they exert a synergistic effect that leads to a more pronounced decrease in MAVS stability (Fig. 6A). This enhanced reduction suggests that the combined action of these two E3 ligases may target MAVS for degradation more effectively than either ligase alone, highlighting the potential for their coordinated roles in regulating protein homeostasis. MARCH5 and Smurf1 are distinct E3 ubiquitin ligases and are classified as RING-type and HECT-type, respectively. They exhibit differences in their structural features, catalytic mechanisms, and substrate specificity (32, 56). Consequently, we hypothesize that upon recruitment by nsp10, these two E3 ligases target MAVS for ubiquitination through distinct catalytic pathways, thereby enhancing its rate of degradation.

The ZBD domain of EAV nsp10 protein contains three structurally different zinc finger motifs, all of which are essential for the replication of the virus (24). In certain proteins, zinc finger motifs have been reported to mediate the dimerization of the protein, such as the C_2_H_2_ zinc finger of transcription factor IIIA (TFIIIA), the C_3_HC_4_ zinc finger of RAG1, and the zinc finger of KSHV ORF57 (38, 39, 58). Previous research has demonstrated that nsp13, the RNA helicase of SARS-CoV-2, can form a dimer to bind the nucleocapsid (59). In this study, we also found that mutations in the zinc finger domain of nsp10 produced two distinct effects: first, one mutation directly weakened its dimerization capacity; second, the other two mutations significantly reduced the abundance of the monomeric form (Fig. 9D). We speculate that the latter phenomenon may be attributable to structural destabilization caused by the mutations, leading to monomer degradation or abnormal aggregation (60). Alternatively, the mutations may have inadvertently introduced new intermolecular interaction interfaces, promoting the formation of higher-order oligomers or aggregates (61). Moreover, we found that the degradation of MAVS by nsp10 depends on the dimerization of nsp10 (Fig. 9C and E). Interestingly, the same 1A domain of nsp10 that associates with MAVS is also required for the interaction of nsp10 with Smurf1 (Fig. 7D and 8C). Analysis of the crystal structure suggests that the dimer of some proteins presents two identical interaction domains away from the dimeric interface (62). We assessed the ability of a monomeric nsp10 mutant to form complexes with MAVS and the E3 ubiquitin ligases Smurf1 and MARCH5. Co-IP results demonstrated that the nsp10 monomeric mutant significantly impaired the interaction between MAVS and Smurf1, while the association between MAVS and MARCH5 remained unaffected (Fig. 9F and G). We speculated that the nsp10 dimer may interact with MAVS and Smurf1 via the same domain as each of the monomers, facilitating interaction between MAVS and Smurf1 by bringing these two proteins closer together. In contrast, MARCH5 interacts with nsp10 through a distinct domain from that of MAVS (Fig. 7D and 8F), and we propose that the formation of the MARCH5-MAVS-nsp10 complex is independent of nsp10 dimerization. Consequently, upon Smurf1 knockout, reduced occupancy of Smurf1 on dimeric nsp10 likely frees up binding sites in the 1A domain, allowing enhanced association between MAVS and nsp10 (Fig. 6I). In contrast, MARCH5 knockout reduces the likelihood of MAVS-nsp10 complex formation, which indicated that MARCH5 may stabilize the MAVS-nsp10 interaction by acting as an auxiliary scaffold (Fig. 6J). Notably, either Smurf1 or MARCH5 depletion diminishes K48-linked ubiquitination of MAVS, resulting in decreased proteasomal degradation of MAVS (Fig. 6D).

In summary, our study provides insights into the potential mechanisms by which EAV nsp10, a nidoviral RNA helicase, inhibits host type I IFN signaling and antiviral responses. We provide compelling evidence that nsp10 recruits the E3 ligases Smurf1 and MARCH5 to facilitate K48-linked polyubiquitination of MAVS, targeting it for degradation (Fig. 11). Our findings, therefore, revealed a new function of EAV RNA helicase and potentially provide the theoretical groundwork for the research and development of clinical drugs.

## MATERIALS AND METHODS

### Cells and viruses

Preparations of eMDMs were obtained from equine peripheral blood mononuclear cells (PBMCs), as described previously, with a minor modification (63). Briefly, PBMCs were isolated from 200 to 300 mL of heparinized horse peripheral blood by centrifugation through a HybriMax Histopaque cushion (density = 1.077 g/cm^3^; Sigma, USA). Isolated PBMCs were washed with RPMI 1640 medium (HyClone, USA) three times and resuspended in RPMI 1640 medium supplemented with 10% horse serum (HyClone, USA). In addition, 10^4^ U/mL penicillin, 10^4^ μg/mL streptomycin, 2 mM L-glutamine, 0.1 mM non-essential amino acids, 1 mM sodium pyruvate, and 0.25 mM sodium HEPES, all of which were purchased from Gibco Corporation (USA), were added to the cultures. These cells were seeded into tissue culture flasks (Corning, USA) at 5 × 10^6^ cells/25 cm^2^ and incubated at 37°C in 5% CO_2_ for approximately 12 h. Non-adherent and loosely adherent cells were removed by mildly shaking the flasks before changing the medium, and the remaining adherent cells were further incubated for 3 days to allow differentiation into eMDMs. HEK293T (ATCC CRL-3216), HEK293 (ATCC CRL-1573), RK-13 (ATCC CCL-37), NBL-6 (horse dermal fibroblast cells, ATCC CCL-57), and HeLa (ATCC CCL-2) cells were maintained in Dulbecco’s modified Eagle medium (HyClone, USA) with 10% fetal bovine serum (FBS) (Sigma, USA), and 1% penicillin and streptomycin (Gibco, USA), and kept at 37°C in 5% CO_2_. A highly cell culture-adapted laboratory variant of the original Bucyrus strain of EAV (ATCC VR-796; Manassas, VA) was kindly gifted by Professor Nianzu Zhang, Key Laboratory of Tropical and Subtropical Animal Virology of the Ministry of Agriculture, China, and was propagated in RK-13 cells in DMEM with 2% FBS at 37°C with 5% CO_2_.

### Antibodies and reagents

The mouse anti-β-actin (A1978), mouse anti-Flag (F1804), and mouse anti-HA (H9658) monoclonal antibodies, as well as the rabbit anti-Flag (F7425) and rabbit anti-HA (H6908) antibodies, were purchased from Sigma-Aldrich. Rat anti-myc (ab206486) antibody was purchased from Abcam (Cambridge, UK). Rat anti-MAVS (14341-1-AP) and rat anti-TOM20 (11802-1-AP) antibodies were purchased from Proteintech. DyLight 800-labeled goat anti-mouse (5230-0415) and DyLight 680-labeled goat anti-rabbit (5230-0403) secondary antibodies were purchased from KPL (USA). Monoclonal antibodies against eqMAVS, nsp10, nsp4, and N were prepared in our laboratory. Briefly, mice were immunized with purified recombinant proteins. Following booster immunizations, splenocytes from immunized mice were isolated and fused with myeloma cells using polyethylene glycol. The resulting hybridoma cells were cultured in selective HAT medium. Supernatants from growing clones were screened for antigen-specific antibody production by enzyme-linked immunosorbent assay (ELISA). Positive clones were subjected to limiting dilution for single-cell subcloning to ensure monoclonality. Selected monoclonal hybridoma lines were expanded, and antibodies were either harvested from culture supernatants or produced in larger quantities by inducing ascites in mice. The proteasome inhibitor MG132 (HY-13259; MCE), lysosome inhibitor CQ (PHR1258; Sigma-Aldrich), and endocytosis inhibitors, including dynasore (HY-15304; MCE) and chlorpromazine (HY-12708; MCE), were used at 20 μM. The autophagy inhibitors 3-MA (HY-19312; MCE) and wortmannin (HY-10197; MCE) were used at 5 mM and 25 nM, respectively.

### Plasmids

The following plasmids were used. The pcDNA3.1-MAVS-HA, pcDNA3.1-MAVS-Flag, pcDNA3.1-huMAVS-Flag, pcDNA3.1-moMAVS-Flag, pcDNA3.1-RNF5-HA, pcDNA3.1-MARCH5-HA, pcDNA3.1-AIP4-HA, pcDNA3.1-MUL1-HA, pcDNA3.1-Smurf1-HA, pcDNA3.1-RNF125-HA, IFN-β-reporter, and Renilla-TK reporter were all stored in our laboratory (64). The ISG54-, ISG56-, NF-κB-, and ISRE-reporters were obtained from Professor Li Huang. The plasmid picEAV is an infectious EAV clone derived from the cell-adapted ppBucyrus strain. This construction contained a human CMV promoter, a HamRz sequence at the 5′ terminus of the viral genome, the 12,704-nucleotide full-length genome of the Bucyrus strain, a poly (A) tail of 69 residues incorporated at the 3′ end of the genome, a HdvRz sequence, and a SV40 late poly (A) at the 3′ end of the construction. This clone was constructed as previously described and kept in our laboratory (65). Genes encoding viral non-structural proteins (nsp3, nsp4, nsp7, nsp9, nsp10, and nsp12) were amplified from picEAV and cloned into the PCAGGS-HA. All expression vectors were generated using the In-Fusion cloning (Clontech, Felicia, CA). The pRK5-HA-ubiquitin-K63 (17606) and pRK5-HA-ubiquitin-K48 (17605) were obtained from Addgene (USA).

### Transfection and luciferase reporter assays

HEK293T cells were seeded in 12-well plates overnight and then transfected with 200 ng IFN, NF-κB, ISRE, ISG54, or ISG56 luciferase reporter (firefly luciferase), 5 ng pRL-TK plasmid (Renilla luciferase), 500 ng Flag-MAVS expressing plasmid, and 500 ng of nsp10-expressing plasmid using polyethyleneimine (PEI) transfection reagent, a cationic polymer prepared by our lab. Twenty-four hours post-transfection, cells were collected, and luciferase activity was measured using a Dual-Luciferase Assay Kit (Promega) on a Synergy2 Reader (Bio-Tek) according to the manufacturer’s protocol. The relative level of gene expression was determined by normalizing firefly luciferase activity to Renilla luciferase activity.

### Real-time PCR

Total RNA was extracted using an RNeasy Mini Kit (Qiagen, Germany) and subjected to reverse transcription using Prime-Script RT reagent kit with a gDNA Eraser (Takara, Japan). Real-time PCR was performed using SYBR-Green (Takara, Japan)-based real-time quantitative PCR analysis on an Agilent Mx3005P. The following primers were used for real-time PCR:

1. IFN-β: F 5′-GTGTTTCTCCACCACGGCTCTTT-3′; R 5′-GACCAATGCAGCA
  TCCTCCTTCT-3′
2. NFκ-B: F 5′-CTGCATCCACAGCTTCCAGAACC-3′; R 5′-CATAGCCGCACC
  GCATTCAGG-3′
3. TNF: F 5′-CCTCAGCCTCTTCTCCTTC-3′; R 5′-TCGGTAACTGCTCTTCCC
  T-3′
4. ISG15: F 5′-GATCGCCCAGAAGACAGGAG-3′; R 5′-CACAGTTCTGCACG
  ACAAGC-3′
5. IL-6: F 5′-GCTGGCTAAGCTGCATTCAC-3′; R 5′-GACCAGAGGAAGGAAT
  GCCC-3′
6. IFIT1: F 5′-TCCTCTCAGCTCCTCAGACC-3′; R 5′-TGAATCTGATCCGC
  GACTGG-3′
7. ACTB: F 5′-CATCTGCTGGAAGGTGGACAA-3′; R 5′-CGACATCCGTAAGG
  ACCTGTA-3′

Relative fold changes in gene expression were determined using the 2^–ΔΔCT^ threshold cycle (CT) method.

### Immunoblotting experiments

Cells were homogenized with lysis buffer (50 mM HEPES-NaOH, pH 7.9; 100 mM NaCl; 50 mM KCl; 0.25% NP-40; 1 mM dithiothreitol) and then centrifuged at 10,000 × *g* for 5 min to remove the cell nuclei. The samples were separated on 12% SDS-PAGE and transferred onto nitrocellulose membranes (Millipore, USA). After blocking with 5% fat-free dry milk (BD, USA) for 2 h at room temperature, the membranes were probed with primary antibodies, followed by secondary antibody. Bands were analyzed by scanning blots using the Odyssey Imaging System (Li-Cor, Lincoln, NE, USA).

### Co-immunoprecipitation (Co-IP) assay

For Co-IP, HEK293T cells were lysed with a cell lysis buffer (150 mM NaCl, 50 mM Tris-HCl, pH 8.0; 5 mM EDTA; 0.5% NP-40) containing 1 mM phenylmethylsulfonyl fluoride and protease inhibitors (Merck-Millipore), followed by incubation for 12 h at 4°C with anti-Flag agarose beads (Thermo Fisher Scientific). Beads were washed four times with lysis buffer, eluted with 1 × SDS loading buffer, and then resolved using SDS-PAGE.

### Immunofluorescence staining

HeLa cells were transfected with the indicated vectors using PEI DNA reagent. HEK293 cells were infected with EAV for 24 h. The cells were fixed with 4% formaldehyde for 30 min, permeabilized with 0.1% Triton X-100 for 15 min at room temperature, and blocked in 5% fat-free milk in PBS for 1 h. Then, the cells were incubated with primary antibody overnight at 4°C. After three washes with PBS, the cells were incubated with Alexa Fluor 488-conjugated goat anti-mouse antibody (Invitrogen, USA), Alexa Fluor 488-conjugated goat anti-rabbit antibody (Invitrogen, USA), Alexa Fluor 555-conjugated goat anti-mouse antibody (Invitrogen, USA), or Alexa Fluor 555-conjugated goat anti-rabbit antibody (Invitrogen, USA) for 1 h at room temperature. The cells were then counterstained with 4,6-diamidino-2-phenylindole (DAPI; Beyotime, China), followed by three additional washes, and were visualized using a confocal fluorescence microscope (Zeiss LSM980 microscopy).

### Establishment of Smurf1 and MARCH5 knockout cell lines

HEK293 cells were used to generate Smurf1 or MARCH5 gene knockout cells using CRISPR-Cas9 technology. The gRNA was designed using the Broad Institute Zhang Lab Guide Design Resources. The sequence targeting Smurf1 was as follows: ATTCGATAACCATTAGCGTG. The sequence targeting MARCH5 was as follows: ATCCACCCAGCGTTGTAGA. DNA fragments that contained the gRNA specific for Smurf1 or MARCH5, a guide RNA scaffold, an RNU6 promoter, and an RNU6 termination signal sequence were synthesized and subcloned into the lentiCRISPRv2 vector. Briefly, HEK293 cells in a 6-well plate were transfected with 1.0 μg of gRNA expression plasmid using PEI DNA reagent. At 24 h post-transfection, cells were treated with puromycin (MCE, HY-K1057) at 0.005 mg/mL for 72 h. The positive clones were validated with DNA sequencing and western blotting.

### RNA interference

Equine MAVS siRNA and control scrambled siRNA were designed and synthesized by Sangon Biotech (China). The sequences targeting MAVS gene were as follows: GAGAUUCUGCCUUACUUGUTT. A total of 2.0 × 10^5^ eMDMs cells were seeded into 6-well plates and cultivated for 24 h, then were transfected with MAVS siRNA or control scrambled siRNA using Lipofectamine RNAiMAX (Invitrogen, USA). Briefly, 100 pmol siRNA in 100 μL serum-free Opti-MEM medium (Gibco, USA) and 6 μL Lipofectamine RNAiMAX in 100 μL of Opti-MEM were mixed and incubated for 5 min at room temperature. The mixtures were then added dropwise to each well.

### Cross-linking assays

HEK-293T cells were collected and washed thrice with ice-cold PBS (pH 8.0). DSS cross-linker (S1885, Sigma) was then added to a final concentration of 250 µM, and the mixture was incubated for 30 min at 37°C. The reaction was quenched with 10 mM Tris-HCl (pH 7.5) for 15 min at room temperature. Cells were subsequently centrifuged at 6,000 × *g* for 15 min at 4°C and lysed with a cell lysis buffer (150 mM NaCl, 50 mM Tris-HCl, pH 8.0; 5 mM EDTA; 0.5% NP-40) containing 1 mM phenylmethylsulfonyl fluoride and protease inhibitors (Merck-Millipore). The cross-linked pellets were resuspended in 100 µL of 1 × SDS loading buffer and then boiled.

### Molecular simulation

The nsp10 structural data were downloaded from the Protein Data Bank (https://www.rcsb.org/structure/4N0N). The eqMAVS, Smurf1, and MARCH5 structural data were downloaded from the AlphaFold Protein Structure Database (https://alphafold.ebi.ac.uk/entry/A0A3Q2ICL5, A0A9L0S8C3, and A0A3Q2IG40). Models of the complexes of nsp10 with MAVS, nsp10 with Smurf1, and nsp10 with MARCH5 were generated using the structural data on the Vakser Lab Server (https://vakserlab.ku.edu/). The results were visualized using PyMOL 2.5.

### Statistical analyses

All statistical analysis was performed in GraphPad Prism version 6.0 (La Jolla, CA, USA). The statistical values were calculated using one-way analysis of variance (ANOVA) or two-tailed Student’s *t*-tests. Each data bar represents the mean value ± SEM (standard error of mean) of at least three independent experiments in all cases. Asterisks indicate the statistical significance: NS, no significance; *P < 0.05; **P < 0.01; ***P < 0.001; ****P < 0.0001.

## Data Availability

The authors confirm that the data supporting the findings of this study are available within the article.

## SUPPLEMENTAL MATERIAL

Fig. S1 jvi.02061-25-s0001.tif EAV nsp4 induces the morphological alterations of mitochondria.

ASM does not own the copyrights to Supplemental Material that may be linked to, or accessed through, an article. The authors have granted ASM a non-exclusive, world-wide license to publish the Supplemental Material files. Please contact the corresponding author directly for reuse.
