# Supplementary figures and images for: Equine arteritis virus Nsp10 promotes MAVS proteasomal degradation via E3 ligases Smurf1/MARCH5
Source: J Virol. 2026 May 19;100(6):e02061-25. doi: 10.1128/jvi.02061-25 (PMC13288486; doi:10.1128/jvi.02061-25)

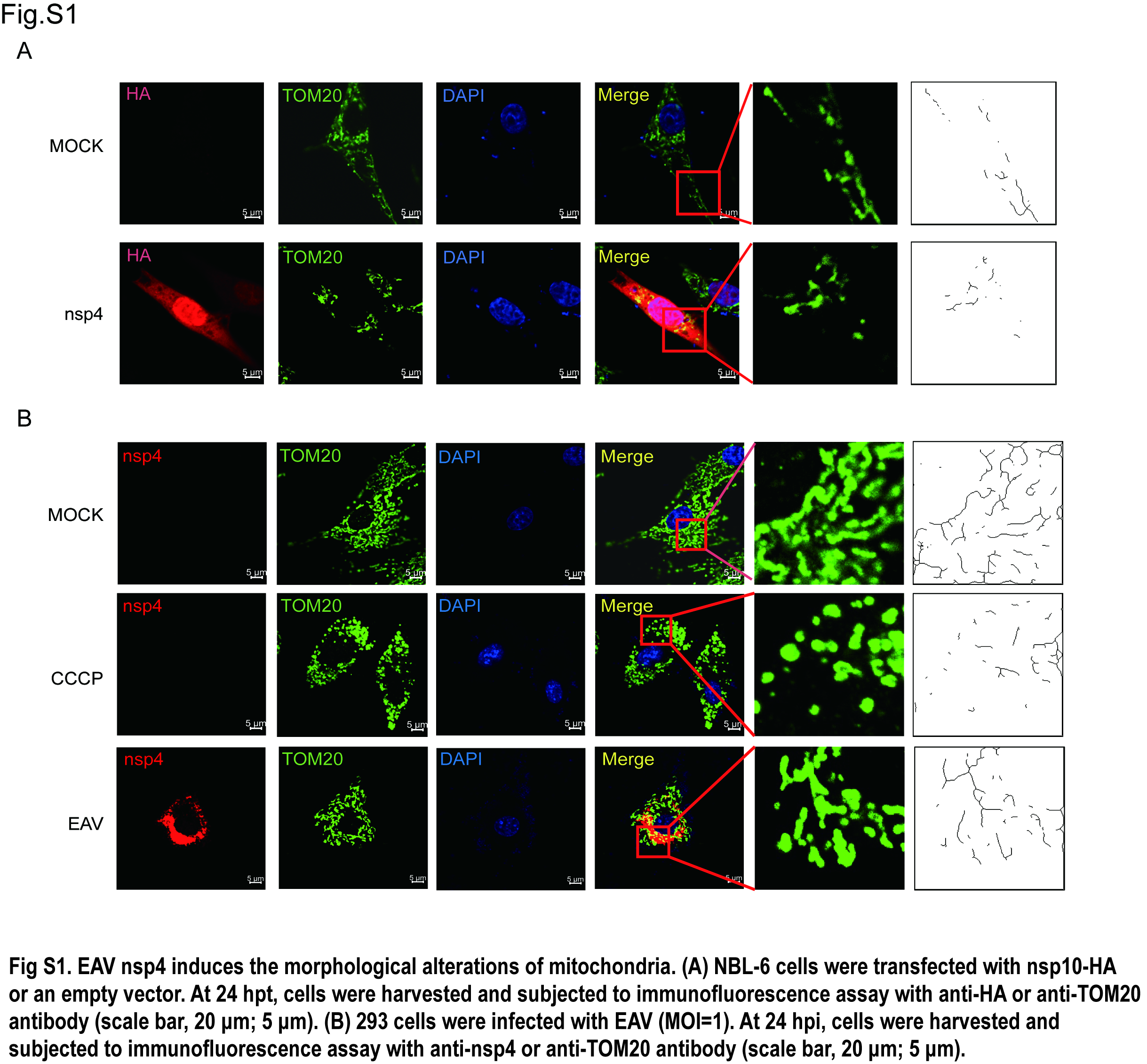

Supplement: Fig. S1 — EAV nsp4 induces the morphological alterations of mitochondria. [file jvi.02061-25-s0001.tif]
